# Flavonoids identified in Australian *Terminalia* inhibit methicillin and β-lactam-resistant pathogens, exhibit efflux pump inhibitory activity, and potentiate conventional antibiotics

**DOI:** 10.1128/spectrum.02374-25

**Published:** 2026-01-16

**Authors:** Muhammad Jawad Yousaf Zai, Matthew James Cheesman, Ian Edwin Cock

**Affiliations:** 1 Centre for Planetary Health and Food Security, Griffith University 5723, Brisbane, Queensland, Australia; 2 School of Environment and Science, Griffith University 95526, Brisbane, Queensland, Australia; 3 School of Pharmacy and Medical Sciences, Griffith University 5723, Southport, Queensland, Australia; University of Calgary, Calgary, Alberta, Canada

**Keywords:** antibiotic resistance, MRSA, ESBL, flavonoid, efflux pump, synergy, combinational therapies, accumulation assay, efflux assay

## Abstract

Plants of the genus *Terminalia* have been widely used in traditional medicine for the treatment of multiple ailments, including infectious diseases. Previously, our group performed metabolomic analyses using liquid chromatography-mass spectrometry of various *Terminalia* spp. and highlighted several phytochemicals (particularly flavonoids) that may contribute to the antibacterial activities of those species. Herein, we screen 15 flavonoids found in *Terminalia* spp. against antibiotic-resistant and antibiotic-sensitive strains of *Escherichia coli*, *Klebsiella pneumoniae*, and *Staphylococcus aureus*. Orientin, hispidulin, vitexin, rutin, fisetin, and isoorientin inhibited the growth of both methicillin- and β-lactam-resistant pathogens, producing MIC values ranging from 250 to 62.5 µg/mL. Orientin and isoorientin were the most effective at restricting the growth of methicillin-resistant and β-lactamase pathogens, with MIC values of 125 µg/mL against *E. coli* and extended-spectrum β-lactamase *E. coli* and 62.5 µg/mL against *S. aureus*. In combination with selected conventional antibiotics, some flavonoids potentiated the antimicrobial activity of selected conventional antibiotics. A total of 4 synergistic, 4 additive, and 22 non-interactive interactions were identified. The toxicity of the flavonoids was examined using *Artemia franciscana* nauplii lethality assays. With the exception of fisetin, genistein, and gossypetin, the flavonoids were non-toxic. Orientin and isoorientin were assessed for their potential to inhibit efflux pumps and demonstrated notable efflux pump inhibitory activity at four different concentrations: 125.0, 62.5, 31.25, and 15.26 µg/mL. The results obtained suggest that these flavonoids could serve as a valuable tool in combating antibiotic resistance.

**IMPORTANCE:**

Bacteria are becoming resistant to many types of antibiotics. This study has identified plant phytochemicals known as flavonoids, which were found to be capable of inhibiting the growth of numerous bacterial pathogens. Evidence is shown which reveals that the compounds are capable of blocking bacterial efflux pumps, which demonstrate that the flavonoids may be valuable compounds in the design of new antibiotic drugs.

## INTRODUCTION

Antibiotics have revolutionized medicine and have facilitated surgeries, safer childbirth, chemotherapy, and organ transplants. However, antimicrobial resistance (AMR) threatens to undermine these achievements. In Europe, AMR is linked to approximately 25,000 deaths each year (1). Similarly, in the USA, AMR pathogens cause approximately 23,000 deaths and over 2 million infections annually (1). The economic impact of AMR in the USA is estimated to be $20 billion in additional medical expenses annually (1). The full extent of AMR worldwide is challenging to measure due to limited epidemiological data, especially in underdeveloped countries. Nonetheless, the available data raise serious concerns. The global emergence of resistance factors such as the plasmid-mediated *bla*_NDM-1_ gene (2), carbapenem-resistant strains of *Klebsiella pneumoniae* (3), and *Escherichia coli* with the *mcr-1* gene (3) demonstrates the risk of AMR and the need to develop novel ways to address this problem. The development of multi-drug antibiotic resistance in the ESKAPE pathogens (*E. coli*, *Staphylococcus aureus*, *K. pneumoniae*, *Acinetobacter baumannii*, *Pseudomonas aeruginosa*, and *Enterobacter* spp.) is particularly concerning, and considerable effort is aimed at developing new and effective antibiotic therapies against these pathogens (4). Therefore, several ESKAPE pathogens were selected for screening in this study.

Traditional medicinal plants have attracted substantial interest in combating AMR in recent years due to their reported antibacterial activities and their potential to potentiate the activity of clinically conventional antibiotics (5). Plants belonging to the genus *Terminalia* have a long history of traditional use to treat various ailments globally, including microbial infections (6). Previously, our group recorded the antimicrobial activity of several *Terminalia* spp., including *Terminalia petiolaris* A. Cunn. ex Benth., *Terminalia canescens* DC. Radlk, *Terminalia ferdinandiana* Exell, *Terminalia microcarpa* Decne, *Terminalia grandiflora* Benth, and *Terminalia muelleri* Benth against a panel of pathogens, including methicillin-resistant and extended-spectrum β-lactamase (ESBL)-expressing strains (7–9). Phytochemical analysis of the *Terminalia* spp. extracts in those studies revealed a range of interesting compounds, including several flavonoids, that may contribute to their antimicrobial activities. Flavonoids belong to a class of natural compounds that have attracted substantial scientific and therapeutic interest due to their reported physiochemical and medicinal properties. Multiple flavonoids have antimicrobial activity and protect plants against pathogens. Additionally, some flavonoids also inhibit the growth of human pathogens and thus have chemotherapeutic potential (10). Interestingly, some plant-derived flavonoids have been reported to inhibit bacterial pathogens through mechanisms distinct from those of conventional antibiotics (11). Therefore, those flavonoids may be effective against bacteria that are otherwise resistant to the actions of conventional antibiotics. Furthermore, it is unlikely that significant bacterial resistance against these compounds currently exists, further highlighting their medicinal potential and making them valuable targets as novel antimicrobial therapies (11).

This study investigated the antibacterial activity of the flavonoids orientin, myricetin, hispidulin, luteolin, vitexin, quercetin, rutin, kaempferol, fisetin, isorhamnetin, gossypetin, isoorientin, apigenin, taxifolin, and genistein, which were highlighted in previous studies due to their presence in the extracts of multiple Australian *Terminalia* spp. possessing strong antibacterial activity (7–9). Herein, we evaluate the activity of the selected flavonoids against *K. pneumoniae* and ESBL-producing *K. pneumoniae*, *E. coli* and ESBL *E. coli*, *Staphylococcus aureus*, and methicillin-resistant *Staphylococcus aureus* (MRSA). These pathogens were selected for screening in this study, as they significantly contribute to infection-related fatalities, and they often exhibit high levels of antibiotic resistance (to multiple classes of antibiotics, in addition to β-lactams). The antimicrobial activity of these flavonoids was also examined in combination with the conventional antibiotics tetracycline, chloramphenicol, ciprofloxacin, gentamicin, and erythromycin to determine if they can enhance their effectiveness, thereby potentially re-purposing those antibiotics for clinical use. The most effective flavonoids in limiting the growth of MRSA and β-lactamase-producing pathogens (orientin and isoorientin) were also evaluated for efflux pump inhibitory activity. The toxicity of flavonoids was determined using *Artemia franciscana* nauplii lethality toxicity assays (*Artemia* lethality assays [ALAs]).

## RESULTS

### Evaluation of MIC

Liquid broth microdilution assays were used to quantify the antimicrobial activity of the flavonoids by determining MICs (Table 1). Orientin was highly effective against *S. aureus* (MIC = 62.5 µg/mL, 140 µM), while isoorientin was the most effective growth inhibitor of *K. pneumoniae* and *S. aureus* (MIC values against both = 62.5 µg/mL, 140 µM). Hispidulin also displayed good activity against *S. aureus* and MRSA (MIC = 125 µg/mL, 416 µM), as did rutin (MIC = 125 µg/mL, 204 µM). Notably, orientin and isoorientin exhibited good activity against both the resistant and sensitive strains of *E. coli* (MIC = 125 µg/mL, 278 µM). Myricetin, luteolin, quercetin, kaempferol, isorhamnetin, gossypetin, apigenin, taxifolin, and genistein failed to inhibit the growth of either the antibiotic-sensitive or resistant strains of *E. coli*, *K. pneumoniae*, and *S. aureus* at 250 µg/mL.

**TABLE 1:** MIC values (µg/mL, µM) of flavonoids and conventional antibiotics against the pathogens tested in this study^*a*^^,^^*b*^

Flavonoids or antibiotics	MIC expressed as µg/mL (µM)					
	*E. coli*	ESBL *E. coli*	*K. pneumoniae*	ESBL *K. pneumoniae*	*S. aureus*	MRSA
Apigenin	–	–	–	–	–	–
Fisetin	250 (873)	–	250 (873)	250 (873)	–	250 (873)
Genistein	–	–	–	–	–	–
Gossypetin	–	–	–	–	–	–
Hispidulin	125 (416)	250 (832)	250 (832)	125 (416)	125 (416)	125 (416)
Isoorientin	125 (278)	125 (278)	125 (278)	**62.5** (140)	**62.5** (140)	125 (278)
Isorhamnetin	–	–	–	–	–	–
Kaempferol	–	–	–	–	–	–
Luteolin	–	–	–	–	–	–
Myricetin	–	–	–	–	–	–
Orientin	125 (278)	125 (278)	125 (278)	125 (278)	**62.5** (140)	125 (278)
Quercetin	–	–	–	–	–	–
Rutin	250 (409)	250 (409)	250 (409)	250 (409)	125 (204)	125 (204)
Taxifolin	–	–	–	–	–	–
Vitexin	250 (578)	250 (578)	250 (578)	250 (578)	125 (290)	250 (578)
Tetracycline	–	–	–	–	1.25 (2.81)	2.5 (5.63)
Chloramphenicol	–	–	–	–	–	–
Ciprofloxacin	0.62 (1.88)	2.5 (7.55)	2.5 (7.55)	2.5 (7.55)	1.25 (3.77)	2.5 (7.55)
Gentamicin	0.31 (0.65)	0.31 (0.65)	0.31 (0.65)	0.31 (0.65)	0.62 (1.30)	0.31 (0.65)
Erythromycin	–	–	–	–	0.625 (0.84)	–
CCCP	3.90 (19)	3.90 (19)	3.90 (19)	7.81 (38)	1.95 (9.5)	3.90 (19)
EtBr	7.81 (19)	7.81 (19)	7.81 (19)	15.61 (40)	3.90 (9.8)	7.81 (19)
Negative control	–	–	–	–	–	–

^*a*^ MIC values of the flavonoids and antibiotic controls represent the mean values of three independent experiments (*n* = 3). MICs are expressed in microgram per milliliter and micromolar. “–” indicates no inhibition was observed at the tested concentration. Highly active MIC values for the flavonoid tests (<100 µg/mL) are highlighted in bold text. CCCP, carbonyl cyanide 3-chlorophenylhydrazone; EtBr, ethidium bromide.

^*b*^ –, no toxicity at all doses tested.

### Fractional inhibitory concentration

All flavonoids that produced MIC values of ≤125 µg/mL against both the resistant and sensitive strains of *E. coli*, *K. pneumoniae*, and *S. aureus* were combined with a range of clinical antibiotics to determine the effects of the flavonoids on the potency of the antibiotics. Orientin and isoorientin each produced MIC values of ≤125 µg/mL (Table 2) and were hence chosen for combinational studies. Multiple classes of interactions were observed for combinations tested against both the resistant and sensitive strains of *E. coli*, *K. pneumoniae*, and *S. aureus* (Table 3). A total of 4 additive, 4 synergistic, and 22 non-interactive interactions were noted, while no antagonistic interactions were apparent.

**TABLE 2:** ∑FIC values for interactions between flavonoids and antibiotics^*a*^^,^*^b^*

Bacterial species	Flavonoid	∑FIC				
		Tetracycline	Chloramphenicol	Ciprofloxacin	Gentamicin	Erythromycin
*E. coli*	Orientin	–	–	1.5	1.25	–
	Isoorientin	–	–	1.5	1.25	–
ESBL *E. coli*	Orientin	–	–	**0.75**	1.25	–
	Isoorientin	–	–	**0.75**	1.25	–
*K. pneumoniae*	Orientin	–	–	1.50	1.25	–
	Isoorientin	–	–	1.50	1.25	–
ESBL *K. pneumoniae*	Orientin	–	–	1.50	1.25	–
	Isoorientin	–	–	2.12	1.50	–
*S. aureus*	Orientin	1.50	–	3	*0.25*	*0.50*
	Isoorientin	1.50	–	3	*0.25*	*0.50*
MRSA	Orientin	1.50	–	**0.75**	1.25	–
	Isoorientin	1.50	–	**0.75**	1.25	–

^*a*^ ∑FIC values of flavonoids in combination with conventional antibiotics against resistant and sensitive strains of *E. coli*,* K. pneumoniae*, and *S. aureus*. Synergy, ≤0.5 (indicated in italic type); additive, >0.5–1.0 (indicated in bold type); indifferent, >1.0–≤4.0; antagonistic, >4.0. FIC values were verified in duplicate experiments. “–” indicates no inhibition at any dose concentration tested.

^*b*^ FIC, fractional inhibitory concentration.

**TABLE 3:** Toxicity (expressed as LC_50_ in µg/mL) and the TI for the flavonoids^*a*^*^,^*^*b*^

Flavonoids	ALA		Therapeutic index					
	LC_50_ value (µg/mL)	Toxicity evaluation	*E. coli*	ESBL *E. coli*	*K. pneumoniae*	ESBL *K. pneumoniae*	*S. aureus*	MRSA
Apigenin	>2,000	Non-toxic	–^*c*^	–	–	–	–	–
Fisetin	>62.5	Toxic	4	–	4	4	–	4
Genistein	>62.5	Toxic	–	–	–	–	–	–
Gossypetin	>125	Toxic	–	–	–	–	–	–
Hispidulin	>2,000	Non-toxic	16	8	8	16	16	16
Isoorientin	>2,000	Non-toxic	16	16	16	32	32	16
Isorhamnetin	>2,000	Non-toxic	–	–	–	–	–	–
Kaempferol	>2,000	Non-toxic	–	–	–	–	–	–
Luteolin	>2,000	Non-toxic	–	–	–	–	–	–
Myricetin	>2,000	Non-toxic	–	–	–	–	–	–
Orientin	>2,000	Non-toxic	16	16	16	16	32	16
Quercetin	>2,000	Non-toxic	–	–	–	–	–	–
Rutin	>2,000	Non-toxic	8	8	8	8	16	16
Taxifoli	>2,000	Non-toxic	–	–	–	–	–	–
Vitexin	>2,000	Non-toxic	8	8	8	8	16	8

^*a*^ ALA, *Artemia *lethality assay; TI, therapeutic index.

^*b*^ TI was determined by LC_50_/MIC.

^*c*^ –, no toxicity at all doses tested.

### Flavonoid antibiotic synergistic interactions at different ratios

**Fig 1:**
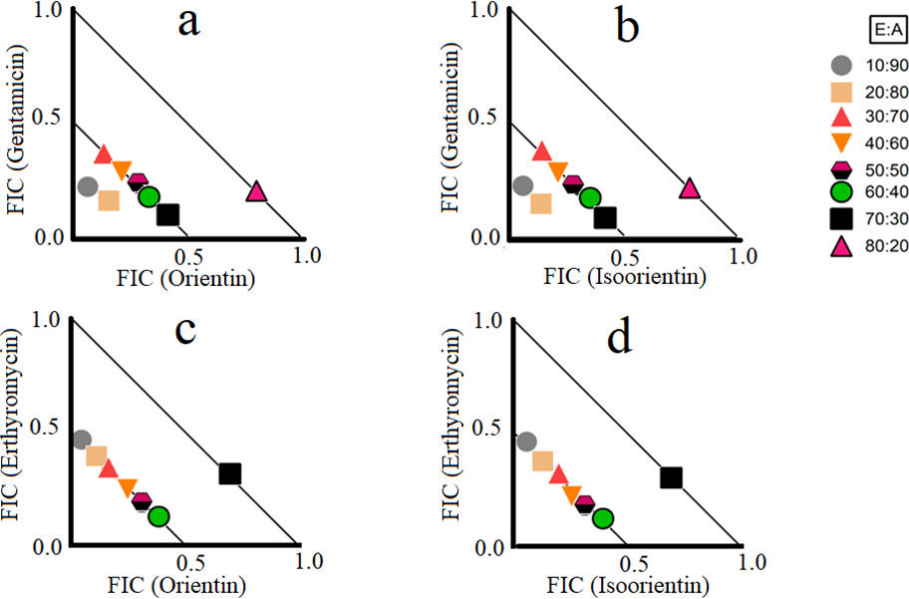
Isobolograms of varying ratios of (**a**) orientin and gentamicin against *S. aureus*, (**b**) isoorientin and gentamicin against *S. aureus*, (**c**) orientin and erythromycin against *S. aureus*, and (**d**) isoorientin and erythromycin against *S. aureus*. Results are displayed as the mean MIC values of two independent experiments. Ratio = % extract:% antibiotic. Ratios ≤0.5/0.5 represent synergy (∑FIC ≤ 0.5). Any ratios >0.5/0.5 and ≤1/1 are considered additive (∑FIC > 0.5–1.0). Only synergistic and additive ratios are depicted in the figure. FIC, fractional inhibitory concentration.

Four flavonoid-antibiotic combinations produced synergistic interactions: orientin with either gentamicin and erythromycin against *S. aureus*, and isoorientin in combination with either gentamicin or erythromycin against *S. aureus*. Therefore, these combinations were tested across various ratios and plotted as isobolograms to identify the ideal ratio(s) at which synergistic interactions occur. Only ratios that produce synergistic and additive effects were included in the isobologram (Fig. 1). The combination of orientin with gentamicin produced a synergistic effect at all ratios containing 10%–70% orientin, while the ratio containing 80% orientin produced an additive effect (Fig. 1a). Combining orientin with erythromycin resulted in synergy at ratios containing 10%–60% orientin, whereas a ratio of 70% orientin and 30% erythromycin showed an additive effect (Fig. 1c). Similarly, isoorientin combined with gentamicin exhibited synergy at ratios containing 10%–60% isoorientin (Fig. 1b), although the ratio containing 70% isoorientin resulted in additive effect. All ratios not included in the isobologram had indifferent effects, indicating no additional benefits compared to using the components individually.

### Ethidium bromide accumulation

**Fig 2:**
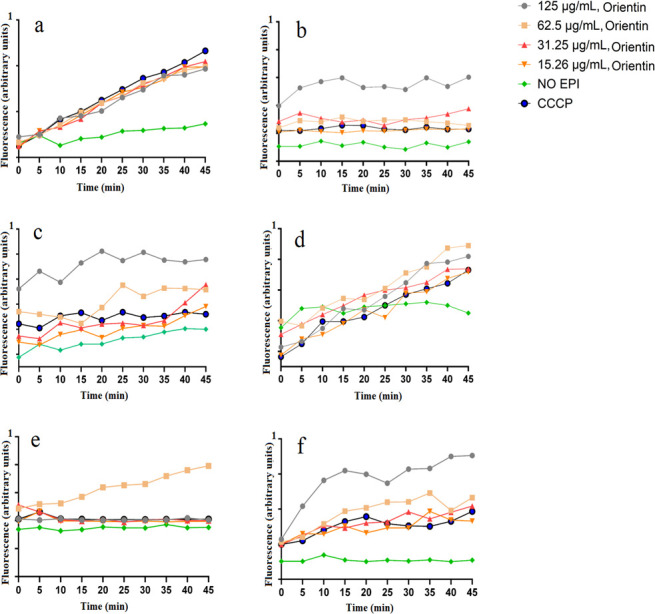
Effect of orientin on the accumulation of ethidium bromide in (**a**) *E. coli*, (**b**) ESBL *E. coli*, (**c**) *K. pneumoniae*, (**d**) ESBL *K. pneumoniae*, (**e**) *S. aureus*, and (**f**) MRSA. Carbonyl cyanide 3-chlorophenylhydrazone (CCCP) was at half of the MIC values.

The effects of the flavonoids MIC of ≤125 µg/mL against the antibiotic-sensitive and the antibiotic-resistant strains of *E. coli*, *K. pneumoniae*, and *S. aureus* on ethidium bromide (EtBr) accumulation were tested against those bacteria. Only two flavonoids (orientin and isoorientin) had MICs of ≤125 µg/mL (Table 2). Four different concentrations of orientin and isoorientin (125.0, 62.50, 31.25, and 15.26 µg/mL) were examined for EtBr accumulation activity in the tested pathogens. Bacterial cultures treated with carbonyl cyanide 3-chlorophenylhydrazone (CCCP) were also tested in parallel as a positive control, while the untreated group with no efflux pump inhibitor (EPI) served as a negative control. The negative control group exhibited the lowest level of EtBr accumulation (Fig. 2), demonstrating that these bacteria have functional efflux pump systems, which reduce intracellular EtBr concentration by pumping it out of the cell. Orientin treatment (125 µg/mL) resulted in the highest EtBr accumulation (compared to the other concentrations and CCCP) in ESBL *E. coli* (Fig. 2b), *K. pneumoniae* (Fig. 2c), and MRSA (Fig. 2f), indicating that it facilitates EtBr uptake and/or inhibits the bacterial efflux mechanisms. In contrast, the greatest EtBr accumulation in *S. aureus* cells was observed when orientin was used at 62.5 µg/mL (Fig. 2e). Notably, orientin and CCCP affected EtBr accumulation by approximately the same amount in *E. coli* (Fig. 2a) and ESBL *K. pneumoniae* (Fig. 2d). These results indicate that orientin significantly increased EtBr accumulation compared to the untreated control (*P* < 0.05) at most time points and at most concentrations (Tables S1 to S6).

**Fig 3:**
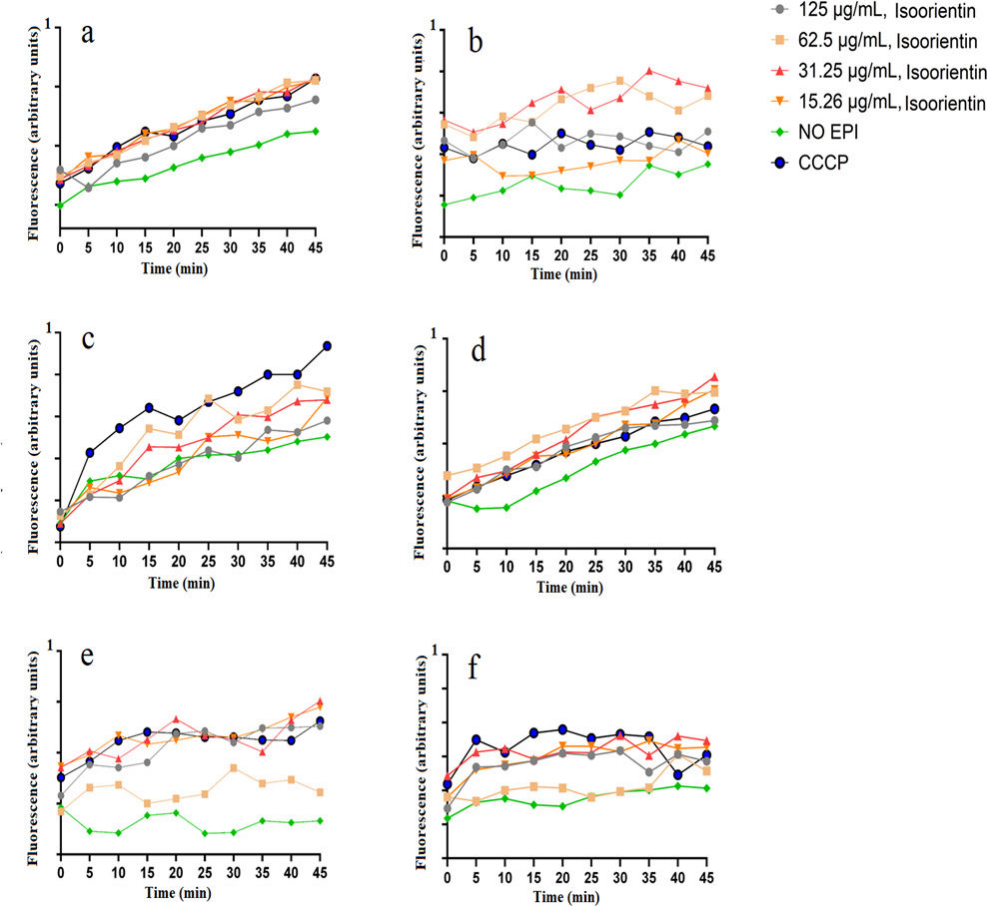
Effect of isoorientin on the accumulation of ethidium bromide in (**a**) *E. coli*, (**b**) ESBL *E. coli*, (**c**) *K. pneumoniae*, (**d**) ESBL *K. pneumoniae*, (**e**) *S. aureus*, and (**f**) MRSA. Carbonyl cyanide 3-chlorophenylhydrazone (CCCP) was at half of the MIC values.

The dose-response effects of isoorientin on EtBr accumulation were evaluated across a range of concentrations from 15.3 to 125 µg/mL, using serial twofold dilutions in antibiotic-sensitive and antibiotic-resistant strains of *E. coli*, *K. pneumoniae*, and *S. aureus* (Fig. 3). Notably, all of the isoorientin concentrations had similar activity in the *E. coli* (Fig. 3a) and ESBL *K. pneumoniae* strains (Fig. 3d). In contrast, ESBL *E. coli* showed the highest fluorescence and therefore the highest EtBr accumulation when they were treated with 62.5 and 31.25 µg/mL of isoorientin (Fig. 3b). These results indicated that isoorientin significantly increased EtBr accumulation compared to the untreated control (*P* <0.05) at most time points and at most concentrations (Tables S7 to S12).

### Evaluation of EtBr efflux

**Fig 4:**
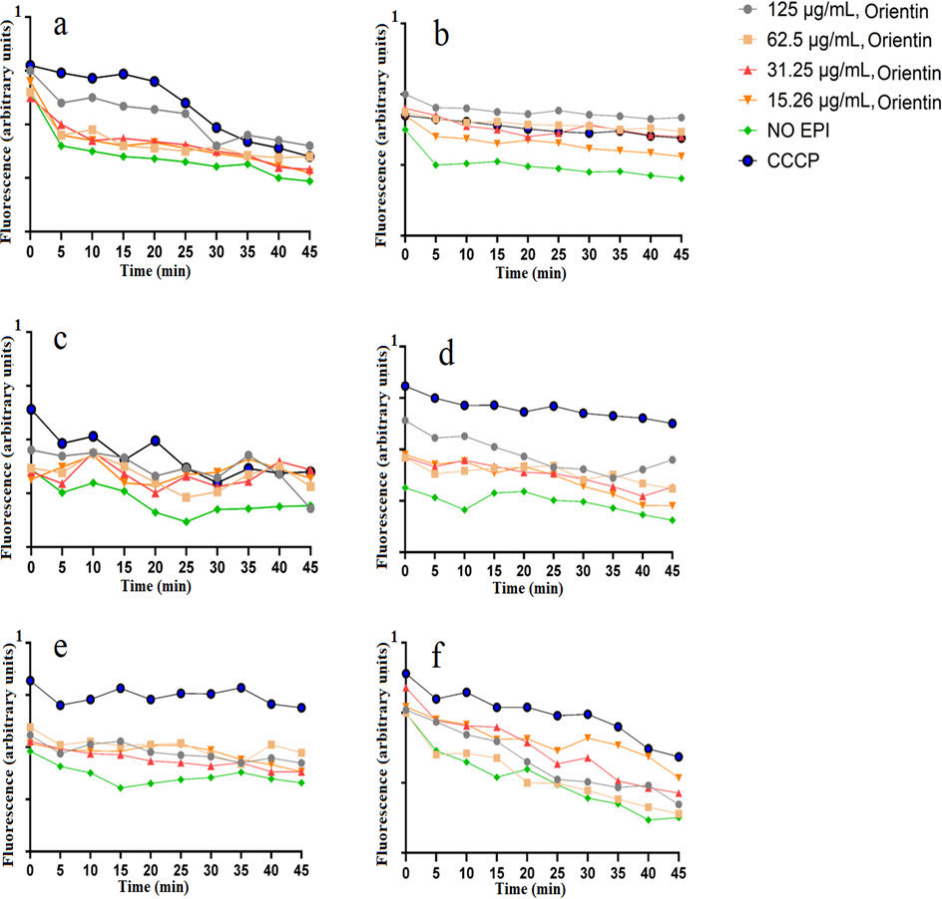
Effects of orientin on the efflux of ethidium bromide in (**a**) *E. coli*, (**b**) ESBL *E. coli*, (**c**) *K. pneumoniae*, (**d**) ESBL *K. pneumoniae*, (**e**) *S. aureus*, and (**f**) MRSA. Carbonyl cyanide 3-chlorophenylhydrazone (CCCP) was at half of the MIC values.

**Fig 5:**
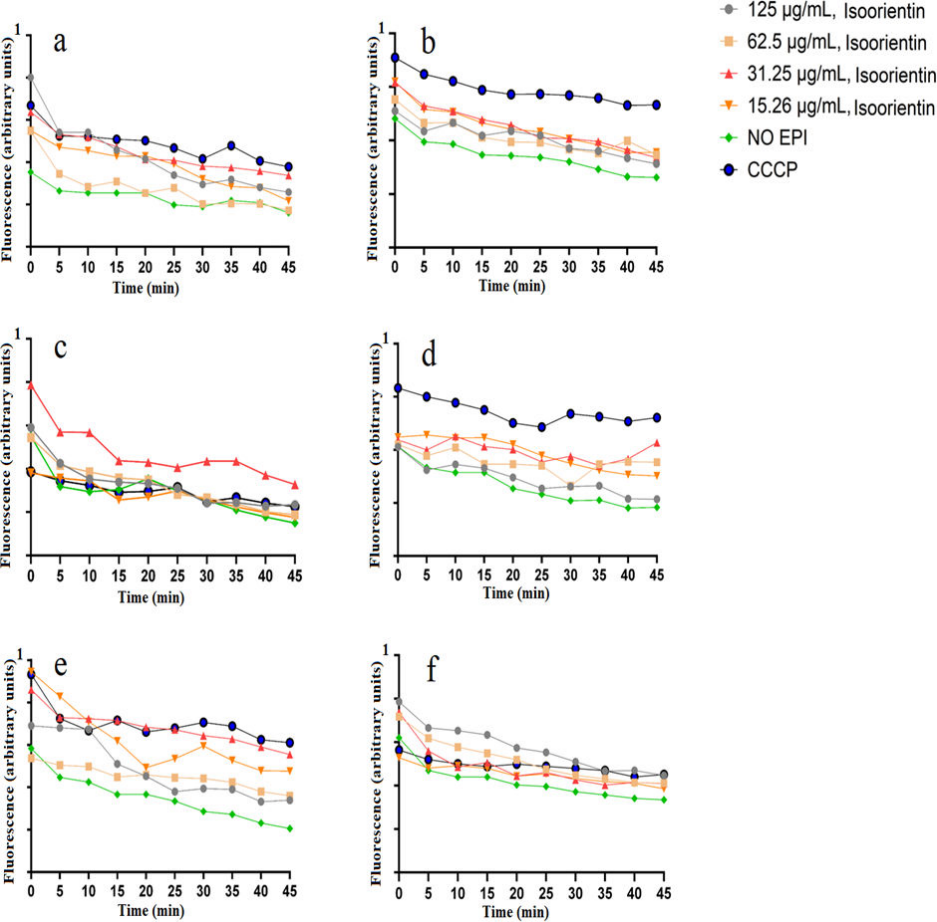
Effect of isoorientin on the efflux of ethidium bromide in (**a**) *E. coli*, (**b**) ESBL *E. coli*, (**c**) *K. pneumoniae*, (**d**) ESBL *K. pneumoniae*, (**e**) *S. aureus*, and (**f**) MRSA. Carbonyl cyanide 3-chlorophenylhydrazone (CCCP) was at half of the MIC values.

The same flavonoids and concentrations tested in the accumulation assays were also tested in the efflux assays to check their ability to inhibit bacterial efflux pumps in sensitive and resistant strains of *E. coli*, *K. pneumoniae*, and *S. aureus*. Among the groups that were treated with different concentrations of orientin, positive control (CCCP), and negative control (no EPI), the EtBr efflux inhibition activity was lowest for the negative control (Fig. 4). Notably, CCCP was a strong inhibitor of the bacterial efflux pump activities. Indeed, CCCP had a higher level of efflux pump inhibitory activity in all of the tested pathogens except ESBL *E. coli,* where the inhibitory activity of 125 µg/mL orientin was slightly greater than the CCCP (Fig. 4b). The result indicated that orientin significantly decreased EtBr efflux compared to the untreated control (*P* <0.05) at most time points and at most concentrations (Tables S13 to S18). The EtBr efflux pump inhibitory activity of 31.25 µg/mL isoorientin against *K. pneumoniae* was slightly higher than the other concentrations (Fig. 5c), while 125 µg/mL isoorientin had better efflux pump inhibitory activity in MRSA (Fig. 5f). The result indicated that isoorientin significantly increased EtBr accumulation compared to the untreated control (*P* < 0.05) at most time points and at most concentrations Tables S19 to S24.

### Toxicity evaluation

The toxicity of the selected flavonoids was evaluated using ALA toxicity assays across a concentration range of 62.5–2,000 µg/mL. The mean % mortality was used to calculate the LC_50_ values (Table 4). Flavonoids that cause <50% mortality at a specific concentration were considered non-toxic at that dosage. For flavonoids that produce >50% mortality, further dilutions were made and tested across various concentrations until a <50% mortality was achieved. All experiments were performed in triplicate (*n* = 3), and results were expressed as mean ± SEM.

**TABLE 4:** Information on the identity, source, and purity of the flavonoids tested in this study

Catalog no.	Flavonoid	Formula	Molecular weight (g/mol)	Purity (%)	Supplier
10010275	Apigenin	C_15_H_10_O_5_	270.2	≥98	Cayman Chemical
A10388	Fisetin	C_15_H_10_O_6_	286.2	>98	Adooq Bioscience
10005167	Genistein	C_15_H_10_O_5_	270.2	≥98	Cayman Chemical
G-500	Gossypetin	C_15_H_10_O_8_	318.24	>93	Indofine Chemical
A13945	Hispidulin	C_16_H_12_O_6_	300.26	>98	Adooq Bioscience
26862	Isoorientin	C_21_H_20_O_11_	448.4	≥95	Cayman Chemical
16496	Isorhamnetin	C_16_H_12_O_7_	316.3	≥98	Cayman Chemical
A10495	Kaempferol	C_15_H_10_O_6_	286.2	>98	Adooq Bioscience
A10541	Luteolin	C_15_H_10_O_6_	286.2	>98	Adooq Bioscience
A10615	Myricetin	C_15_H_10_O_8_	318.2	>98	Adooq Bioscience
A12096	Orientin	C_21_H_20_O_11_	448.38	>98	Adooq Bioscience
A10766	Quercetin	C_15_H_10_O_7_	302.2	>98	Adooq Bioscience
A10815	Rutin	C_27_H_30_O_16_	610.5	>98	Adooq Bioscience
18647	Taxifolin	C_15_H_12_O_7_	304.3	≥98	Cayman Chemical
A12135	Vitexin	C_21_H_20_O_10_	432.4	>98	Adooq Bioscience

### Therapeutic index calculation

To further assess the safety of the flavonoids as therapeutic agents, their therapeutic indexes (TIs) were determined (Table 4). In this study, TI values >4 were considered noteworthy. The safety of hispidulin, isoorientin, orientin, rutin, and vitexin was especially encouraging as they exhibited TI values substantially greater than 4 against all tested bacterial strains. Therefore, these flavonoids were deemed safe for therapeutic use *in vitro* and therefore potential druggable targets. However, additional cell line toxicity evaluations and *in vivo* studies are necessary to validate their safety before they can be considered for clinical application.

## DISCUSSION

Previously, our group reported the antimicrobial activity of various *Terminalia* spp., and subsequent liquid chromatography-mass spectrometry (LC-MS) analysis identified and highlighted several phytochemicals (particularly flavonoids) that may contribute to the antibacterial activity of those *Terminalia* spp. (7–9). In this study, the 15 flavonoid compounds: orientin (Fig. S1a), myricetin (Fig. S1b), hispidulin (Fig. S1c), luteolin (Fig. S1d), vitexin (Fig. S1e), quercetin (Fig. S1f), rutin (Fig. S1g), kaempferol (Fig. S1h), fisetin (Fig. S1i), isorhamnetin (Fig. S1j), gossypetin (Fig. S1k), apigenin (Fig. S1l), taxifolin (Fig. S1m), genistein (Fig. S1n), and isoorientin (Fig. S1o) were examined for antimicrobial activities against the antibiotic-sensitive and antibiotic-resistant strains of *E. coli*, *K. pneumoniae*, and *S. aureus*. The antimicrobial activity of the flavonoids was initially examined at a concentration of 250 µg/mL, and the potency was quantified by further screening across a range of concentrations. Orientin and isoorientin exhibited strong activity against *S. aureus* (MIC = 62.5 µg/mL; Table 2). Isoorientin was also highly active against *K. pneumoniae* (MIC = 62.5 µg/mL). Myricetin, quercetin, kaempferol, gossypetin, apigenin, and taxifolin failed to inhibit the growth of the antibiotic-sensitive and resistant strains of *E. coli*, *K. pneumoniae*, and *S. aureus* when tested at 250 µg/mL. Previous studies have reported the antibacterial activity of apigenin and quercetin against *E. coli* and *S. aureus*, albeit at a very high concentration (12). Indeed, that study noted that the MIC of apigenin against *E. coli* was 7578.45 µM, while for *S. aureus*, the MIC exceeded 7,578.45 µM (13). Similarly, the MIC of quercetin against *E. coli* was reported to be >3,388.04 and 13,552.14 µM against S. *aureus* in that study. Those MIC values would generally be considered ineffective, particularly for pure compounds. In our study, the starting concentration of apigenin tested was 925 µM, while quercetin was tested at 827 µM. This may account for the fact that we failed to observe any inhibitory activity in our study, and inhibition may have been noted if we screened at higher concentrations. However, as noted above, higher MIC values such as these indicate low (or no) activity and therefore are misleading.

Another study tested the antimicrobial activity of rutin against three different strains of *E. coli* (NEC-01, NEC-03, and NEC-04) and MRSA (NSA-02, NSA-06, and NSA-08) isolated from the fast food (13). The reported MIC values against those *E. coli* strains ranged from 400 to 1200 µg/mL, while the MICs against the MRSA strains ranged from 800 to 1600µg/mL (12). Similar (although lower) MIC values were noted against different strains of these bacteria. In our study, the MIC of rutin against *E. coli* was determined to be 250 µg/mL, while it was 125 µg/mL against MRSA. Our study used reference strains of *E. coli* and MRSA, which have well-reported antibiotic-susceptibility profiles, whereas the earlier study used strains that were not well characterized. The strains used in the earlier study may have been more highly antibiotic resistant or may have incorporated more than one resistance mechanism, which may account for the higher MICs reported in earlier studies. However, the susceptibility of those bacteria was not well defined, and the flavonoids should be evaluated in future studies against an extended panel of bacteria that incorporates multiple strains of each bacterium.

Flavonoids exert antimicrobial properties through mechanisms distinct from those of conventional antibiotic drugs, making them potentially valuable for enhancing antibacterial therapies. Flavonoids can compromise membrane integrity, leading to metabolic dysfunction, ultimately resulting in bacterial death (14). The antibacterial effects of the flavonoid catechin are primarily due to its interactions with the cell membrane. Catechins have been shown to disrupt bacterial membranes by binding to the lipid bilayer and inhibiting or inactivating both intracellular and extracellular enzymes (15). Apigenin, acacetin, rhamnetin, and morin also destabilize membrane structures by disrupting and disorienting membrane lipids, leading to leakage from vesicles (16). Flavonoids have also been reported to inhibit the formation of bacterial biofilms. Notably, bacterial biofilm-based infections account for a substantial proportion of all microbial and chronic infections in both humans and animals, as well as contributing to food spoilage (17). A key characteristic of bacteria growing as biofilms is their increased resistance to antimicrobial agents, which is frequently 10–1,000 times greater than that of their planktonic counterparts (18). Isovitexin significantly inhibits biofilm activity in *S. aureus* (19), while epicatechin (at 2%–15%) decreases biofilm formation by 55%–66% (20). Quorum sensing has been identified as a key regulatory factor in biofilm production for *Salmonella* Typhimurium, *Vibrio* species, and *E. coli* (21). Kaempferol, apigenin, naringenin, and quercetin are effective inhibitors of cell-to-cell signaling required for biofilm formation (21). Quercetin also increases the expression of several iron siderophore proteins, reducing the availability of Fe^3+^, which is essential for biofilm formation in *Pseudomonas aeruginosa* (22).

Flavonoids have also been reported to possess significant DNA gyrase (also known as topoisomerase IV) inhibition activity. Notably, DNA gyrase is a vital enzyme for DNA replication and is unique to prokaryotes, making it an appealing target for antibacterial drugs. Apigenin and quercetin inhibit DNA gyrase activity in *E. coli*, thereby inhibiting their replication (23). Quercetin can also target the subunit B of DNA gyrase in *Mycobacterium tuberculosis* and *Mycobacterium smegmatis* (24). Kaempferol and chrysin were also shown to inhibit DNA gyrase in *E. coli*, while myricetin was reported to be a substantially less effective inhibitor (25). Luteolin and its structurally related flavonols (including myricetin and morin) inhibit the replicative helicases RecBCD and DnaB helicase/nuclease in *E. coli* (26). Flavonoids also inhibit the electron transport chain and ATP synthesis and are therefore also promising targets for the development of novel antibiotic therapies. Furthermore, the treatment of *S. aureus* with 6-prenylapigenin and isobavachalcone derived from *Dorstenia* species caused depolarization of the bacterial membrane (27).

Synergistic combination therapies represent a promising area of medical research aimed at combating antibiotic-resistant bacteria (5). In this study, we observed 22 non-interactive, 4 synergistic, and 4 additive interactions (Table 3). Synergistic combinations greatly enhance the antimicrobial efficacy of antibiotics compared to additive interactions, offering significant potential for the development of novel and highly effective antibiotic therapies. Synergistic interactions were observed when orientin or isoorientin were combined with gentamicin and erythromycin and tested against *S. aureus* (Fig. 1). Orientin and isoorientin may block the resistance mechanism of gentamicin and erythromycin, although this needs to be verified in future studies. In contrast, non-interactive combinations do not enhance or diminish antimicrobial effects compared to the pure compound or antibiotic used individually, suggesting they are safe for simultaneous use, although they offer no added benefits. Notably, flavonoids and antibiotic synergistic interactions have also been reported in previous studies. Apigenin exhibits synergistic interactions with ceftazidime against ceftazidime-resistant *Enterobacter cloacae* (28). Apigenin also reverses antibiotic activity against quinolone-resistant *S. aureus* bacteria (29). A DNA cleavage assay revealed that apigenin inhibited a resistance gene encoding a modified DNA gyrase responsible for quinolone resistance but did not affect the wild-type DNA gyrase gene, which remains sensitive to levofloxacin (29). Additional studies have reported synergistic interactions between apigenin and levofloxacin or gentamicin against *P. aeruginosa* and MRSA (30). Synergistic interactions of gentamicin with apigenin, epigallocatechin-3-gallate, and luteolin against *E. coli*, *K. pneumoniae*, *P. aeruginosa*, and *S. aureus* have also been reported (30). However, data on the antibacterial activity of orientin and isoorientin and their interaction with antibiotics are limited compared to other flavonoids. Importantly, the selected flavonoids were non-toxic in the *Artemia* nauplii assay, except for fisetin, genistein, and gossypetin (Table 4). Notably, TI values were evaluated for several flavonoids. Indeed, TI values greater than 4 were determined for hispidulin, isoorientin, orientin, rutin, and vitexin. The calculated TI values for isoorientin against ESBL *K. pneumoniae* (31) and *S. aureus* (31) and for orientin against *S. aureus* (31) were particularly noteworthy and indicated that these combinations may be safe for therapeutic use. However, further validation of flavonoids’ safety for medicinal use requires testing them against a broad range of human cell lines to confirm their low toxicity.

In this study, we have also evaluated the efflux pump inhibitory activity of orientin and isoorientin. Orientin and isoorientin had better antimicrobial activity against the sensitive and resistant strains of *E. coli*, *K. pneumoniae*, and *S. aureus*. This made them promising candidates for further investigation to determine whether their antibacterial effects were linked to efflux pump inhibition. We performed an EtBr efflux assay to check for the efflux pump inhibitory activity. The use of EtBr to confirm efflux pump inhibition is well established and widely reported (32). EtBr can intercalate with bacterial DNA, consequently leading to cellular death. To counteract this effect, bacterial efflux proteins expel EtBr from the cell (33). Substances that inhibit efflux pumps enhance the retention of EtBr, antibiotics, and other toxic compounds within the cell, thereby decreasing the survival of bacteria (31). Orientin and isoorientin were found to enhance EtBr accumulation in both antibiotic-sensitive and antibiotic-resistant strains of *E. coli*, *K. pneumoniae*, and *S. aureus* in comparison to the negative control (no EPI) (Fig. 2 and 3). Orientin (125 µg/mL) exhibited potent efflux pump inhibitory activity in ESBL *E. coli* (Fig. 2b), *K. pneumoniae* (Fig. 2c), and MRSA (Fig. 2f). Orientin at 62.5 µg/mL exhibited strong efflux pump inhibitory activity in *S. aureus* (Fig. 2e). Similarly, isoorientin at 62.5 and 31.25 µg/mL also demonstrated efflux pump inhibitory activity in ESBL *E. coli*, with these concentrations showing slightly greater activity compared to others (Fig. 3b). Notably, little difference was seen in the efflux pump inhibitory activity between different concentrations of isoorientin when tested against the other pathogens screened in this study (Fig. 3). In the EtBr efflux assay, bacteria preloaded with EtBr revealed that orientin (31.25 µg/mL) showed the greatest ability to maintain fluorescence intensity in *K. pneumoniae* (Fig. 5c). Initially, fluorescence intensity was highest when bacteria were loaded with EtBr, but over 45 min, it gradually decreased as efflux pumps reduced the intracellular EtBr concentration. Flavonoids have been extensively reported to possess efflux pump inhibitory activity (31), with apigenin specifically known to exhibit such activity (34). In this study, a fluorescence-based assay was used to assess the efflux pump inhibitory activity of the selected flavonoids. However, these assays are prone to optical matrix interference, such as quenching of EtBr fluorescence, which may affect the accuracy of efflux pump activity measurements (35). To mitigate this issue, future studies should also utilize LC-MS to quantify EtBr concentrations. The LC-MS method allows precise measurement of extracellular EtBr levels without relying on fluorescence. Notably, the concentration-response relationship observed using the LC-MS is inverse to fluorescence-based measurements, as higher inhibitor concentrations trap EtBr within bacterial cells, leading to a decrease in extracellular EtBr levels detected by LC-MS (35). Additionally, microscopic imaging studies may also provide additional detail about the efflux pump inhibition mechanism, and future studies should incorporate such evaluations.

Future studies should also explore whether the flavonoids examined in this study exhibit antimicrobial activity through other mechanisms, including (i) inhibition of protein synthesis, (ii) interference with cell wall synthesis, (iii) disruption of metabolic pathways, (iv) interference with nucleic acid synthesis, (v) inhibition of membrane function, and (vi) impairment of membrane function. Furthermore, once there is a greater understanding of the effects and mechanisms of orientin and isoorientin, *in vivo* studies using rodent models will be required to further evaluate the effects of these compounds, as well as to examine the biodynamic and biokinetic properties of these compounds.

## MATERIALS AND METHODS

### Materials

All solvents used in this study were of analytical grade (AR) and purchased from Ajax Fine-Chemicals Ltd, Australia. Flavonoids were procured from Sapphire Biosciences, Australia, and their technical information is listed in Table 4. Mueller-Hinton media (broth and agar) were obtained from Oxoid Ltd, Australia. The components of the phosphate-buffered saline (PBS) were 0.0027 M potassium chloride, 0.01 M phosphate buffer, and 0.137 M sodium chloride (pH 7.4). Unless otherwise specified, all other chemicals and reagents were AR and were purchased from Sigma-Aldrich, Australia.

### Bacterial strains

The antimicrobial effects of the flavonoids were examined against MRSA and ESBL-resistant bacterial strains, as well as their antibiotic-susceptible counterparts. ESBL *K. pneumoniae* (ATCC 700603) and MRSA (ATCC 43300) were acquired from the American Type Culture Collection (ATCC). An ESBL *E. coli* strain was provided by the Gold Coast University Hospital (Southport, Australia). These strains’ susceptibilities to multiple antibiotics have previously been verified in our laboratory (6–8). Antibiotic-sensitive strains of *K. pneumoniae* (ATCC 31488), *S. aureus* (ATCC 25923), and *E. coli* (ATCC 25922) were purchased from ATCC and included in this study for comparison. All bacterial strains were stored as glycerol stocks at −30°C until use.

### Bacterial culture growth

To ensure pure cultures for the screening studies, individual stock solutions of the bacterial pathogens were streaked onto freshly prepared Mueller-Hinton agar plates and incubated for 24 h at 37°C to obtain pure cultures. A single colony was subsequently transferred to 50 mL of sterile Muller-Hinton broth and incubated at 37°C until the bacteria reached the log growth phase (as determined by absorbance at 600 nm), except for MRSA, which was incubated at 35°C. Following the incubation, the purity of each of the cultures was verified by re-streaking them individually onto fresh Mueller-Hinton agar plates.

### Liquid microdilution assays

Flavonoids were dissolved in sterile autoclaved water containing 2.5% dimethyl sulfoxide (DMSO). The MIC of each flavonoid was determined against each bacterium at an initial concentration of 250 µg/mL (and one in two serial dilutions of the initial concentration) using standardized liquid-phase microdilution assays (7). The concentration of DMSO in the first well tested was 0.6%. The experiment was performed in triplicate (*n* = 3) using standard methods (7), and the results are expressed as means ± SEM.

### Flavonoids: antibiotic combinational effects and identification of optimal ratio

The interaction between flavonoids and conventional antibiotics was first assessed at a 1:1 ratio. The same general protocol that was outlined for the liquid microdilution assays (7) was also used to determine the MICs of each component in the combinations. Fractional inhibitory concentration (FIC) was then evaluated using the following formulas:

FIC (flavonoid) = (MIC of flavonoid in combination) / MIC of flavonoid alone

FIC (antibiotic) = (MIC of antibiotic in combination) / MIC of antibiotic alone

∑FIC = FIC (flavonoid) + FIC (antibiotic)

∑FIC values ≤0.5 were categorized as synergistic; >0.5–≤1.0 were designated as additive; >1.0–≤4.0 were termed as non-interactive; and >4.0 were classed as antagonistic.

Combinations that produced synergistic interactions were further evaluated across a number of different ratios, and the results were used to identify combination ratios that were synergistic. All combinations were tested across ratios containing 10%–90% flavonoid component % (with corresponding reciprocal percentages of antibiotic component), using 10% increasing increments. The measured FIC values were used to plot isobolograms, which were used to determine the synergistic flavonoid and antibiotic ratios.

### EtBr accumulation assay

EtBr accumulation assays were performed using standard procedures (36). Briefly, log-stage bacterial cultures in Mueller-Hinton broth were prepared by incubation at 37°C until an optical density of 0.8 was attained when the absorbance was measured at 600 nm (OD_600_). The bacterial culture was then sedimented by centrifugation for 3 min at 13,000 rpm. The supernatant was aspirated and discarded, and the pellet was washed and re-suspended in 0.25 M PBS (pH 7.4). The OD_600_ of the bacterial culture was adjusted to 0.4, and glucose and EtBr were added to 1 mL of bacterial culture to give final concentrations of 0.4% (w/v) and 1 µg/mL respectively. A 95 µL volume of bacterial culture and 5 µL of the test compounds at four different concentrations (125 µg/mL, 62.5 µg/mL, 31.25 µg/mL, and 15.26 µg/mL) were aspirated into individual wells of black flat-bottomed 96 well plates. PBS was used as a negative control, while CCCP was tested in parallel at half the concentration of the MIC determined for each bacterial species (Table 2) as a positive control. The fluorescence was measured using excitation and emission wavelengths of 530 nm 600 nm at 5-min intervals for 45 min using a Molecular Devices, Spectra Max M3 plate reader placed in Griffith University, Brisbane, Australia. Each experiment was performed in triplicate (*n* = 3), and the results are expressed as the mean ± SEM.

### EtBr efflux assay

The effect of the flavonoid test compounds on EtBr efflux was measured using standard methods (35). Briefly, EtBr accumulation was observed at 25°C in the absence of glucose. The concentration of EtBr in each assay was adjusted to a concentration equivalent to half of its MIC concentration (Table 2) for each bacterium to ensure optimal accumulation, while maintaining cellular viability. Following this incubation, the EtBr-loaded cells were sedimented by centrifugation at 13,000 rpm for 3 min. The cells were subsequently resuspended in 0.25 M PBS buffer (pH 7.4) containing 0.4% glucose, without EtBr. The OD_600_ was adjusted to 0.4, and 95 µL aliquots were added to individual wells of black flat-bottom 96-well plates. To initiate the assay, 5 µL of each of the potential EPIs was added to individual wells. Replica tubes without any EPIs served as negative controls, while cells exposed to CCCP were included as positive controls. Fluorescence (excitation and emission wavelengths of 530 and 600 nm) was measured at 5-min intervals for 45 min using a Molecular Devices Spectra Max M3 plate reader at Griffith University, Brisbane, Australia. Each experiment was performed in triplicate (*n* = 3) and expressed as mean ± SEM.

### Toxicity studies

The toxicity of all flavonoids was determined using standard ALAs (7) across a concentration range of 62.5–2,000 µg/mL. Positive controls (400 µL of potassium dichromate at a test concentration of 1 mg/mL) were included on all plates, along with wells containing 400 µL of artificial seawater (Red Sea Pharm Ltd, Pituach, Israel) as a negative control. The experiment was performed in triplicate (*n* = 3), and the results were expressed as mean ± SEM.

### Calculation of the therapeutic index

The TI of the flavonoids was determined using the following formula and included as an indicator of their potential for therapeutic application:

Therapeutic index = (ALA LC_50_) / (MIC)

### Statistical analysis

The ALA toxicity and ethidium bromide assays were performed three times, with internal triplicates evaluated for each independent experiment (*n* = 9). The results are presented as the mean ± SEM. Statistical differences between control and test groups were analyzed using one-way ANOVA, with *P* values <0.05 considered statistically significant. Liquid microdilution assays were conducted on two consecutive days, each with internal replicates (*n* = 4), to confirm the reproducibility of the results.

### Conclusion

The need to combat AMR has resulted in a significant rise in research investigating natural products as potential sources for new antibiotic treatment. In this study, we examined the antimicrobial activity of different flavonoids against the sensitive and resistant strains of *E. coli*, *K. pneumoniae*, and *S. aureus*. Orientin, hispidulin, vitexin, rutin, fisetin, and isoorientin inhibited the activity of the antibiotic-resistant ESBL and MDR pathogens as effectively as for their antibiotic-sensitive counterparts. Thus, these flavonoids may have novel and/or unstudied antibacterial mechanisms. Orientin and isoorientin were also evaluated for their ability to inhibit efflux pumps and were found to exhibit efflux pump inhibitory activity. Future studies should explore whether these flavonoids possess distinct antimicrobial mechanisms and evaluate their potential as effective antimicrobial agents. The toxicity of these compounds should be tested against a wide range of human cell lines to confirm their toxicity and safety for medicinal use.

## Data Availability

All data are available from the corresponding author on reasonable request.

## SUPPLEMENTAL MATERIAL

Supplemental material spectrum.02374-25-s0001.docx Tables S1 to S24; Fig. S1.

ASM does not own the copyrights to Supplemental Material that may be linked to, or accessed through, an article. The authors have granted ASM a non-exclusive, world-wide license to publish the Supplemental Material files. Please contact the corresponding author directly for reuse.

## References

[1] Dadgostar P. 2019. Antimicrobial resistance: implications and costs. Infect Drug Resist 12:3903–3910. doi:10.2147/IDR.S234610 31908502 PMC6929930

[2] Yong D, Toleman MA, Giske CG, Cho HS, Sundman K, Lee K, Walsh TR. 2009. Characterization of a new metallo-beta-lactamase gene, bla_NDM-1_, and a novel erythromycin esterase gene carried on a unique genetic structure in Klebsiella pneumoniae sequence type 14 from India. Antimicrob Agents Chemother 53:5046–5054. doi:10.1128/AAC.00774-09 19770275 PMC2786356

[3] Liu YY, Wang Y, Walsh TR, Yi LX, Zhang R, Spencer J, Doi Y, Tian GB, Dong BL, Huang XH, Yu LF, Gu DX, Ren HW, Chen XJ, Lv LC, He DD, Zhou HW, Liang ZS, Liu JH, Shen JZ. 2016. Emergence of plasmid-mediated colistin resistance mechanism MCR-1 in animals and human beings in China: a microbiological and molecular biological study. Lancet Infect Dis 16:161–168. doi:10.1016/S1473-3099(15)00424-7 26603172

[4] De Oliveira DMP, Forde BM, Kidd TJ, Harris PNA, Schembri MA, Beatson SA, Paterson DL, Walker MJ. 2020. Antimicrobial resistance in ESKAPE pathogens. Clin Microbiol Rev 33:10–128. doi:10.1128/CMR.00181-19 PMC7227449 32404435

[5] Cheesman MJ, Ilanko A, Blonk B, Cock IE. 2017. Developing new antimicrobial therapies: are synergistic combinations of plant extracts/compounds with conventional antibiotics the solution? Pharmacogn Rev 11:57–72. doi:10.4103/phrev.phrev_21_17 28989242 PMC5628525

[6] Cock IE. 2015. The medicinal properties and phytochemistry of plants of the genus Terminalia (Combretaceae). Inflammopharmacology 23:203–229. doi:10.1007/s10787-015-0246-z 26226895

[7] Zai MJ, Cheesman MJ, Cock IE. 2023. Terminalia petiolaris A.Cunn ex Benth. extracts have antibacterial activity and potentiate conventional antibiotics against β-lactam-drug-resistant bacteria. Antibiotics (Basel) 12:1643. doi:10.3390/antibiotics12111643 37998845 PMC10669112

[8] Zai MJ, Cheesman MJ, Cock IE. 2024. Selected Australian Terminalia species extracts inhibit β-lactam drug-resistant bacteria growth and potentiate the activity of conventional antibiotics: bioactivities and phytochemistry. Microorganisms 12:498. doi:10.3390/microorganisms12030498 38543548 PMC10972150

[9] Zai MJ, Cheesman MJ, Cock IE. 2024. Phytochemical evaluation of Terminalia canescens DC. Radlk. extracts with antibacterial and antibiotic potentiation activities against selected β-lactam drug-resistant bacteria. Molecules 29:1385. doi:10.3390/molecules29061385 38543020 PMC10974770

[10] Wen WW, Alseekh S, Fernie AR. 2020. Conservation and diversification of flavonoid metabolism in the plant kingdom. Curr Opin Plant Biol 55:100–108. doi:10.1016/j.pbi.2020.04.004 32422532

[11] Harborne JB, Williams CA. 2000. Advances in flavonoid research since 1992. Phytochemistry 55:481–504. doi:10.1016/S0031-9422(00)00235-1 11130659

[12] Al-Shabib NA, Husain FM, Ahmad I, Khan MS, Khan RA, Khan JM. 2017. Rutin inhibits mono and multi-species biofilm formation by foodborne drug resistant Escherichia coli and Staphylococcus aureus. Food Control 79:325–332. doi:10.1016/j.foodcont.2017.03.004

[13] Yan Y, Xia X, Fatima A, Zhang L, Yuan G, Lian F, Wang Y. 2024. Antibacterial activity and mechanisms of plant flavonoids against Gram-negative bacteria based on the antibacterial statistical model. Pharmaceuticals (Basel) 17:292. doi:10.3390/ph17030292 38543078 PMC10974178

[14] Hartmann M, Berditsch M, Hawecker J, Ardakani MF, Gerthsen D, Ulrich AS. 2010. Damage of the bacterial cell envelope by antimicrobial peptides gramicidin S and PGLa as revealed by transmission and scanning electron microscopy. Antimicrob Agents Chemother 54:3132–3142. doi:10.1128/AAC.00124-10 20530225 PMC2916356

[15] Reygaert WC. 2014. The antimicrobial possibilities of green tea. Front Microbiol 5:434. doi:10.3389/fmicb.2014.00434 25191312 PMC4138486

[16] Ollila F, Halling K, Vuorela P, Vuorela H, Slotte JP. 2002. Characterization of flavonoid--biomembrane interactions. Arch Biochem Biophys 399:103–108. doi:10.1006/abbi.2001.2759 11883909

[17] Jamal M, Ahmad W, Andleeb S, Jalil F, Imran M, Nawaz MA, Hussain T, Ali M, Rafiq M, Kamil MA 2. 2018. Bacterial biofilm and associated infections. J Chin Med Assoc 81:7–11. doi:10.1016/j.jcma.2017.07.012 29042186

[18] Kon K, Rai M. 2016. Antibiotic resistance: mechanisms and new antimicrobial approaches. 1st edition, p 1–413. Academic Press.

[19] Awolola GV, Koorbanally NA, Chenia H, Shode FO, Baijnath H. 2014. Antibacterial and anti-biofilm activity of flavonoids and triterpenes isolated from the extracts of Ficus sansibarica Warb. subsp. sansibarica (Moraceae) extracts. Afr J Tradit Complement Altern Med 11:124–131. doi:10.4314/ajtcam.v11i3.19 25371574 PMC4202430

[20] El-Adawi H, El-Deeb N. 2012. Inhibitory effect of grape seed extract (GSE) on cariogenic bacteria. Planta Med 78:1288–1288. doi:10.1055/s-0032-1321341

[21] Vikram A, Jayaprakasha GK, Jesudhasan PR, Pillai SD, Patil BS. 2010. Suppression of bacterial cell-cell signalling, biofilm formation and type III secretion system by citrus flavonoids. J Appl Microbiol 109:515–527. doi:10.1111/j.1365-2672.2010.04677.x 20163489

[22] Ouyang J, Sun F, Feng W, Sun Y, Qiu X, Xiong L, Liu Y, Chen Y. 2016. Quercetin is an effective inhibitor of quorum sensing, biofilm formation and virulence factors in Pseudomonas aeruginosa. J Appl Microbiol 120:966–974. doi:10.1111/jam.13073 26808465

[23] Plaper A, Golob M, Hafner I, Oblak M, Šolmajer T, Jerala R. 2003. Characterization of quercetin binding site on DNA gyrase. Biochem Biophys Res Commun 306:530–536. doi:10.1016/S0006-291X(03)01006-4 12804597

[24] Suriyanarayanan B, Shanmugam K, Santhosh RS. 2013. Synthetic quercetin inhibits mycobacterial growth possibly by interacting with DNA gyrase. Rom Biotech Lett 18:8587–8593.

[25] Wu T, Zang X, He M, Pan S, Xu X. 2013. Structure-activity relationship of flavonoids on their anti-Escherichia coli activity and inhibition of DNA gyrase. J Agric Food Chem 61:8185–8190. doi:10.1021/jf402222v 23926942

[26] Xu H, Ziegelin G, Schröder W, Frank J, Ayora S, Alonso JC, Lanka E, Saenger W. 2001. Flavones inhibit the hexameric replicative helicase RepA. Nucleic Acids Res 29:5058–5066. doi:10.1093/nar/29.24.5058 11812837 PMC97556

[27] Dzoyem JP, Hamamoto H, Ngameni B, Ngadjui BT, Sekimizu K. 2013. Antimicrobial action mechanism of flavonoids from Dorstenia species. Drug Discov Ther 7:66–72. doi:10.5582/ddt.2013.v7.2.66 23715504

[28] Eumkeb G, Chukrathok S. 2013. Synergistic activity and mechanism of action of ceftazidime and apigenin combination against ceftazidime-resistant Enterobacter cloacae. Phytomedicine 20:262–269. doi:10.1016/j.phymed.2012.10.008 23218402

[29] Morimoto Y, Baba T, Sasaki T, Hiramatsu K. 2015. Apigenin as an anti-quinolone-resistance antibiotic. Int J Antimicrob Agents 46:666–673. doi:10.1016/j.ijantimicag.2015.09.006 26526895

[30] Hanci H, Igan H. 2023. Antimicrobial synergistic effects of apigenin, (-)-epigallocatechin-3-gallate, myricetin and luteolin in combination with some antibiotics. Ann Agric Environ Med 30:61–64. doi:10.26444/aaem/161220 36999857

[31] Patel D, Kosmidis C, Seo SM, Kaatz GW. 2010. Ethidium bromide MIC screening for enhanced efflux pump gene expression or efflux activity in Staphylococcus aureus. Antimicrob Agents Chemother 54:5070–5073. doi:10.1128/AAC.01058-10 20855743 PMC2981236

[32] Viveiros M, Martins A, Paixão L, Rodrigues L, Martins M, Couto I, Fähnrich E, Kern WV, Amaral L. 2008. Demonstration of intrinsic efflux activity of Escherichia coli K-12 AG100 by an automated ethidium bromide method. Int J Antimicrob Agents 31:458–462. doi:10.1016/j.ijantimicag.2007.12.015 18343640

[33] Banerjee A, Majumder P, Sanyal S, Singh J, Jana K, Das C, Dasgupta D. 2014. The DNA intercalators ethidium bromide and propidium iodide also bind to core histones. FEBS Open Bio 4:251–259. doi:10.1016/j.fob.2014.02.006 PMC3958746 24649406

[34] Brown AR, Ettefagh KA, Todd D, Cole PS, Egan JM, Foil DH, Graf TN, Schindler BD, Kaatz GW, Cech NB. 2015. A mass spectrometry-based assay for improved quantitative measurements of efflux pump inhibition. PLoS One 10:e0124814. doi:10.1371/journal.pone.0124814 25961825 PMC4427306

[35] Hudson SA, Ecroyd H, Kee TW, Carver JA. 2009. The thioflavin T fluorescence assay for amyloid fibril detection can be biased by the presence of exogenous compounds. FEBS J 276:5960–5972. doi:10.1111/j.1742-4658.2009.07307.x 19754881

[36] Rodrigues L, Wagner D, Viveiros M, Sampaio D, Couto I, Vavra M, Kern WV, Amaral L. 2008. Thioridazine and chlorpromazine inhibition of ethidium bromide efflux in Mycobacterium avium and Mycobacterium smegmatis. J Antimicrob Chemother 61:1076–1082. doi:10.1093/jac/dkn070 18310137

