# Supplementary material for: Flavonoids identified in Australian Terminalia inhibit methicillin and β-lactam-resistant pathogens, exhibit efflux pump inhibitory activity, and potentiate conventional antibiotics
Source: Microbiol Spectr. 2026 Jan 16;14(3):e02374-25. doi: 10.1128/spectrum.02374-25 (PMC12955415; doi:10.1128/spectrum.02374-25)
Supplement: Supplemental material — Tables S1 to S24; Fig. S1. [file spectrum.02374-25-s0001.docx]

Supplementary data

**Antimicrobial activity of pure flavonoids identified in Australian *Terminalia* spp. against methicillin and β-lactam resistant pathogens**

Muhammad Jawad Yousaf Zai ^1,2^, Matthew James Cheesman ^3^, Ian Edwin Cock ^1,2*^

^1^ Centre for Planetary Health and Food Security, Griffith University, Brisbane, QLD 4111,

^2^ School of Environment and Science, Griffith University, Brisbane, QLD 4111, Australia

^3^ School of Pharmacy and Medical Sciences, Griffith University, Southport, QLD 4222, Australia

^*^ Correspondence: I.Cock@griffith.edu.au; Tel.: +6173735763

| Supp. Table 1: Effect of different concentrations of orientin on the accumulation of ethidium bromide in *E. coli* | | | | | | | | | | | | |
| --- | --- | --- | --- | --- | --- | --- | --- | --- | --- | --- | --- | --- |
| Time  hh:mm:ss | 125 µg/mL | | 62.5 µg/mL | | 31.25 µg/mL | | 15.26 µg/mL | | NO EPI | | CCCP | |
| 0:00:00 | 84497 | 84401 | 83316 | 83322 | 82409 | 82590 | 82301 | 82206 | 84398 | 81808 | 82399 | 82542 |
| 0:05:00 | 85022 | 85153 | 84886 | 84837 | 85635 | 85759 | 85843 | 85809 | 84995 | 84583 | 84740 | 84932 |
| 0:10:00 | 88567 | 88599 | 87101 | 87134 | 86633 | 86655 | 86499 | 86562 | 82506 | 82756 | 88290 | 88270 |
| 0:15:00 | 89055 | 89202 | 89792 | 89698 | 88359 | 88331 | 89015 | 89392 | 84091 | 84089 | 89901 | 89984 |
| 0:20:00 | 90128 | 90135 | 91902 | 91989 | 91876 | 91803 | 91590 | 91723 | 84183 | 84656 | 92276 | 92464 |
| 0:25:00 | 93057 | 93078 | 93464 | 93318 | 93320 | 93430 | 93521 | 94988 | 85736 | 85639 | 94701 | 94819 |
| 0:30:00 | 94769 | 94799 | 96263 | 96256 | 95925 | 95978 | 94993 | 95602 | 85790 | 85998 | 97189 | 97266 |
| 0:35:00 | 97846 | 97853 | 97360 | 97371 | 97659 | 97601 | 96945 | 96953 | 86323 | 86289 | 98378 | 98574 |
| 0:40:00 | 98024 | 98078 | 99019 | 99025 | 99730 | 99745 | 99790 | 99711 | 86056 | 86808 | 100653 | 100723 |
| 0:45:00 | 99336 | 99341 | 99791 | 99890 | 100927 | 100937 | 99745 | 99731 | 87382 | 87303 | 103199 | 103243 |
| Positive control = carbonyl cyanide 3-chlorophenylhydrazone (CCCP), Negative control = No efflux pump inhibitor (NO EPI), an experiment is performed in duplicate (*n*=2) | | | | | | | | | | | | |

| Supp. Table 2: Effect of different concentrations of orientin on the accumulation of ethidium bromide in ESBL *E. coli* | | | | | | | | | | | | |
| --- | --- | --- | --- | --- | --- | --- | --- | --- | --- | --- | --- | --- |
| Time  hh:mm:ss | 125 µg/mL | | 62.5 µg/mL | | 31.25 µg/mL | | 15.26 µg/mL | | NO EPI | | CCCP | |
| 0:00:00 | 24841 | 24953 | 21191 | 20804 | 21417 | 22843 | 19948 | 20553 | 17754 | 17584 | 20461 | 20643 |
| 0:05:00 | 28010 | 28160 | 23788 | 20789 | 20844 | 26457 | 20296 | 20756 | 17357 | 17988 | 20578 | 20397 |
| 0:10:00 | 29194 | 29287 | 22895 | 21240 | 20949 | 24433 | 20258 | 20380 | 18276 | 18926 | 21193 | 20510 |
| 0:15:00 | 29847 | 29967 | 24580 | 21275 | 21322 | 22738 | 19963 | 20302 | 17650 | 17962 | 20947 | 21950 |
| 0:20:00 | 28190 | 28231 | 23352 | 20913 | 20999 | 23725 | 20536 | 20420 | 18421 | 18379 | 21034 | 21790 |
| 0:25:00 | 28298 | 28372 | 23387 | 21397 | 20552 | 22362 | 20414 | 20375 | 17959 | 17124 | 20880 | 20639 |
| 0:30:00 | 27763 | 27859 | 23675 | 21200 | 21079 | 23820 | 20610 | 20539 | 17175 | 17110 | 20719 | 20528 |
| 0:35:00 | 29989 | 29957 | 22809 | 21418 | 20803 | 24763 | 20726 | 20871 | 18330 | 18242 | 21786 | 20438 |
| 0:40:00 | 28339 | 28413 | 22306 | 21397 | 20390 | 26553 | 20811 | 20518 | 17986 | 17031 | 20744 | 20713 |
| 0:45:00 | 30198 | 30091 | 21594 | 21244 | 20863 | 27989 | 20504 | 21111 | 18611 | 18390 | 20712 | 20834 |
| Positive control = carbonyl cyanide 3-chlorophenylhydrazone (CCCP), Negative control = No efflux pump inhibitor (NO EPI), an experiment is performed in duplicate (*n*=2) | | | | | | | | | | | | |

| Supp. Table 3: Effect of different concentrations of orientin on the accumulation of ethidium bromide in *S. aureus* | | | | | | | | | | | | |
| --- | --- | --- | --- | --- | --- | --- | --- | --- | --- | --- | --- | --- |
| Time  hh:mm:ss | 125 µg/mL | | 62.5 µg/mL | | 31.25 µg/mL | | 15.26 µg/mL | | NO EPI | | CCCP | |
| 0:00:00 | 20650 | 20518 | 24221 | 24325 | 31008 | 20234 | 19751 | 19980 | 16894 | 16930 | 20260 | 20845 |
| 0:05:00 | 20398 | 19986 | 25790 | 25882 | 20023 | 26191 | 20032 | 26491 | 17451 | 17677 | 20753 | 25499 |
| 0:10:00 | 20828 | 20605 | 26267 | 26060 | 20931 | 19808 | 19848 | 19886 | 16345 | 16394 | 20363 | 20883 |
| 0:15:00 | 20345 | 20125 | 28459 | 28510 | 19733 | 19808 | 19944 | 19780 | 16967 | 16683 | 20249 | 20736 |
| 0:20:00 | 20074 | 20318 | 31327 | 32494 | 20401 | 20488 | 19904 | 19549 | 17673 | 17791 | 20264 | 20401 |
| 0:25:00 | 20159 | 20261 | 32674 | 32673 | 19489 | 19443 | 19518 | 19679 | 17416 | 17403 | 20247 | 20552 |
| 0:30:00 | 19944 | 20530 | 33001 | 33089 | 20296 | 20144 | 20116 | 19642 | 17641 | 17121 | 19826 | 20888 |
| 0:35:00 | 20418 | 20192 | 36570 | 35325 | 20819 | 20292 | 19697 | 19422 | 18636 | 18585 | 20244 | 20645 |
| 0:40:00 | 20161 | 21749 | 37470 | 38624 | 20446 | 19661 | 19667 | 19783 | 17509 | 17411 | 20203 | 20764 |
| 0:45:00 | 20331 | 20176 | 39246 | 39960 | 20314 | 19638 | 19725 | 19811 | 17334 | 17869 | 20194 | 20834 |
| Positive control = carbonyl cyanide 3-chlorophenylhydrazone (CCCP), Negative control = No efflux pump inhibitor (NO EPI), an experiment is performed in duplicate (*n*=2) | | | | | | | | | | | | |

| Supp. Table 4: Effect of different concentrations of orientin on the accumulation of ethidium bromide in MRSA | | | | | | | | | | | | |
| --- | --- | --- | --- | --- | --- | --- | --- | --- | --- | --- | --- | --- |
| Time  hh:mm:ss | 125 µg/mL | | 62.5 µg/mL | | 31.25 µg/mL | | 15.26 µg/mL | | NO EPI | | CCCP | |
| 0:00:00 | 25653 | 25715 | 25289 | 25324 | 24929 | 24998 | 24584 | 25420 | 22201 | 22957 | 24698 | 25250 |
| 0:05:00 | 29943 | 30895 | 25804 | 25998 | 26191 | 26202 | 26390 | 26491 | 22677 | 22460 | 25234 | 25499 |
| 0:10:00 | 34289 | 34104 | 27998 | 27902 | 27156 | 28176 | 26567 | 26452 | 23759 | 23172 | 26320 | 27730 |
| 0:15:00 | 35521 | 35484 | 29667 | 29710 | 27339 | 27312 | 27593 | 27590 | 22831 | 22672 | 28350 | 28103 |
| 0:20:00 | 34870 | 34962 | 30198 | 30182 | 27390 | 28648 | 26698 | 26612 | 22895 | 22167 | 28600 | 29255 |
| 0:25:00 | 33589 | 33692 | 30834 | 30991 | 28233 | 28190 | 27027 | 27615 | 22687 | 22781 | 27170 | 28675 |
| 0:30:00 | 35719 | 35698 | 31099 | 31032 | 29611 | 29605 | 27276 | 27313 | 22659 | 22553 | 27689 | 27613 |
| 0:35:00 | 35791 | 35800 | 32105 | 32296 | 28789 | 28609 | 29778 | 29679 | 22637 | 22911 | 27447 | 27549 |
| 0:40:00 | 37560 | 37504 | 31131 | 28471 | 29601 | 29548 | 28331 | 28441 | 22506 | 22529 | 28216 | 28316 |
| 0:45:00 | 37677 | 37634 | 31707 | 31628 | 30826 | 30048 | 28199 | 28297 | 22938 | 22541 | 30002 | 29351 |
| Positive control = carbonyl cyanide 3-chlorophenylhydrazone (CCCP), Negative control = No efflux pump inhibitor (NO EPI), an experiment is performed in duplicate (*n*=2) | | | | | | | | | | | | |

| Supp. Table 5: Effect of different concentrations of orientin on the accumulation of ethidium bromide in *K. pneumoniae* | | | | | | | | | | | | |
| --- | --- | --- | --- | --- | --- | --- | --- | --- | --- | --- | --- | --- |
| Time  hh:mm:ss | 125 µg/mL | | 62.5 µg/mL | | 31.25 µg/mL | | 15.26 µg/mL | | NO EPI | | CCCP | |
| 0:00:00 | 20911 | 20313 | 19774 | 19634 | 18699 | 18730 | 18401 | 18487 | 17819 | 17880 | 19201 | 19217 |
| 0:05:00 | 21299 | 21315 | 19730 | 19468 | 18693 | 18553 | 18278 | 18374 | 18415 | 18389 | 19099 | 19050 |
| 0:10:00 | 20776 | 20875 | 19835 | 19130 | 19309 | 19259 | 18698 | 18791 | 18178 | 18167 | 19586 | 19535 |
| 0:15:00 | 21388 | 21910 | 19199 | 19232 | 19084 | 19027 | 18905 | 18976 | 18419 | 18408 | 19677 | 19662 |
| 0:20:00 | 22210 | 22118 | 19845 | 19887 | 19221 | 19204 | 18697 | 18666 | 18467 | 18409 | 19475 | 19227 |
| 0:25:00 | 21699 | 21743 | 20702 | 20758 | 19267 | 19250 | 19054 | 19034 | 18609 | 18652 | 19914 | 19452 |
| 0:30:00 | 22182 | 21960 | 20256 | 20313 | 19199 | 19152 | 19158 | 19152 | 18636 | 18691 | 19442 | 19470 |
| 0:35:00 | 21666 | 21760 | 20599 | 20642 | 19473 | 19218 | 19187 | 19118 | 18876 | 18891 | 19501 | 19530 |
| 0:40:00 | 21868 | 21512 | 20571 | 20626 | 20099 | 20061 | 19599 | 19543 | 19045 | 19026 | 19632 | 19678 |
| 0:45:00 | 21941 | 21626 | 20914 | 20256 | 20701 | 20783 | 19919 | 19909 | 19009 | 19001 | 19601 | 19602 |
| Positive control = carbonyl cyanide 3-chlorophenylhydrazone (CCCP), Negative control = No efflux pump inhibitor (NO EPI), an experiment is performed in duplicate (*n*=2) | | | | | | | | | | | | |

| Supp. Table 6: Effect of different concentrations of orientin on the accumulation of ethidium bromide in ESBL *K. pneumoniae* | | | | | | | | | | | | |
| --- | --- | --- | --- | --- | --- | --- | --- | --- | --- | --- | --- | --- |
| Time  hh:mm:ss | 125 µg/mL | | 62.5 µg/mL | | 31.25 µg/mL | | 15.26 µg/mL | | NO EPI | | CCCP | |
| 0:00:00 | 40202 | 40274 | 41913 | 41963 | 41187 | 41108 | 39751 | 39744 | 41212 | 41877 | 39667 | 39638 |
| 0:05:00 | 40665 | 40648 | 41643 | 41664 | 41733 | 41765 | 40620 | 40991 | 42677 | 42917 | 40487 | 40485 |
| 0:10:00 | 41293 | 41706 | 42834 | 42830 | 42301 | 42395 | 41191 | 41009 | 42993 | 42901 | 41993 | 41949 |
| 0:15:00 | 42734 | 42789 | 43404 | 43452 | 42911 | 42982 | 41833 | 41824 | 42445 | 42481 | 41947 | 41956 |
| 0:20:00 | 42622 | 42698 | 43337 | 43382 | 43675 | 43668 | 42499 | 42945 | 42956 | 42900 | 42263 | 42240 |
| 0:25:00 | 43532 | 43586 | 44103 | 44100 | 44003 | 44000 | 42299 | 42201 | 43004 | 43000 | 42938 | 42999 |
| 0:30:00 | 44453 | 44477 | 45114 | 45113 | 44198 | 44155 | 43743 | 43792 | 43132 | 43100 | 43767 | 43706 |
| 0:35:00 | 45529 | 45937 | 45588 | 45500 | 44504 | 44500 | 43876 | 43889 | 43289 | 43200 | 44059 | 44085 |
| 0:40:00 | 45876 | 45828 | 46734 | 46738 | 45387 | 45352 | 44689 | 44829 | 43067 | 43000 | 44467 | 44436 |
| 0:45:00 | 46102 | 46196 | 46967 | 46901 | 45332 | 45382 | 45193 | 45170 | 42534 | 42500 | 45333 | 45307 |
| Positive control = carbonyl cyanide 3-chlorophenylhydrazone (CCCP), Negative control = No efflux pump inhibitor (NO EPI), an experiment is performed in duplicate (*n*=2) | | | | | | | | | | | | |

| Supp. Table 7: Effect of different concentrations of isoorientin on the accumulation of ethidium bromide in *E. coli* | | | | | | | | | | | | |
| --- | --- | --- | --- | --- | --- | --- | --- | --- | --- | --- | --- | --- |
| Time  hh:mm:ss | 125 µg/mL | | 62.5 µg/mL | | 31.25 µg/mL | | 15.26 µg/mL | | NO EPI | | CCCP | |
| 0:00:00 | 47933 | 47960 | 47064 | 47818 | 47181 | 47170 | 47098 | 47039 | 44961 | 44940 | 46871 | 46828 |
| 0:05:00 | 46482 | 46392 | 48658 | 48491 | 48367 | 48312 | 49076 | 49065 | 46526 | 46591 | 48016 | 48099 |
| 0:10:00 | 48589 | 48527 | 4974 | 49213 | 49566 | 49549 | 49545 | 49018 | 46901 | 46974 | 49858 | 49872 |
| 0:15:00 | 49056 | 49025 | 50561 | 50324 | 50181 | 50887 | 51005 | 51022 | 47292 | 47236 | 51167 | 51178 |
| 0:20:00 | 49923 | 49989 | 51563 | 51557 | 51031 | 51638 | 51474 | 51420 | 48138 | 48155 | 50849 | 50816 |
| 0:25:00 | 51464 | 51472 | 52519 | 52505 | 51934 | 51912 | 52785 | 52381 | 48938 | 48973 | 52025 | 52129 |
| 0:30:00 | 51767 | 51740 | 53536 | 53363 | 53434 | 53616 | 53783 | 53777 | 49442 | 49518 | 52771 | 52703 |
| 0:35:00 | 52829 | 52868 | 54148 | 54250 | 54249 | 54818 | 53745 | 53756 | 50038 | 50106 | 53898 | 53888 |
| 0:40:00 | 53272 | 53092 | 55383 | 55329 | 54597 | 54543 | 54947 | 54953 | 50948 | 50989 | 54211 | 54202 |
| 0:45:00 | 53873 | 53889 | 55337 | 55709 | 55734 | 55775 | 55582 | 55522 | 51122 | 51315 | 55687 | 55670 |
| Positive control = carbonyl cyanide 3-chlorophenylhydrazone (CCCP), Negative control = No efflux pump inhibitor (NO EPI), an experiment is performed in duplicate (*n*=2) | | | | | | | | | | | | |

| Supp. Table 8: Effect of different concentrations of isoorientin on the accumulation of ethidium bromide in ESBL *E. coli* | | | | | | | | | | | | |
| --- | --- | --- | --- | --- | --- | --- | --- | --- | --- | --- | --- | --- |
| Time  hh:mm:ss | 125 µg/mL | | 62.5 µg/mL | | 31.25 µg/mL | | 15.26 µg/mL | | NO EPI | | CCCP | |
| 0:00:00 | 11671 | 11666 | 11804 | 11861 | 11900 | 11928 | 11401 | 11424 | 10971 | 10808 | 11503 | 11579 |
| 0:05:00 | 11401 | 11453 | 11777 | 11707 | 11589 | 11943 | 11487 | 11490 | 10993 | 10975 | 11439 | 11451 |
| 0:10:00 | 11515 | 11723 | 11932 | 11954 | 11817 | 11918 | 11297 | 11237 | 11019 | 11059 | 11684 | 11627 |
| 0:15:00 | 11810 | 11958 | 11891 | 11886 | 12128 | 12121 | 11257 | 11243 | 11260 | 11218 | 11474 | 11495 |
| 0:20:00 | 11485 | 11669 | 12232 | 12098 | 12234 | 12281 | 11393 | 11300 | 11034 | 11085 | 11745 | 11752 |
| 0:25:00 | 11538 | 11959 | 12319 | 12300 | 12099 | 12035 | 11328 | 11354 | 11067 | 11060 | 11621 | 11614 |
| 0:30:00 | 11649 | 11778 | 12392 | 12388 | 12214 | 12146 | 11439 | 11425 | 11010 | 11007 | 11548 | 11552 |
| 0:35:00 | 11689 | 11601 | 12221 | 12200 | 12599 | 12510 | 11483 | 11420 | 11333 | 11394 | 11774 | 11768 |
| 0:40:00 | 11599 | 11526 | 12038 | 12029 | 12065 | 12702 | 11638 | 11672 | 11122 | 11388 | 11747 | 11704 |
| 0:45:00 | 11744 | 11806 | 12227 | 12205 | 12206 | 12392 | 11583 | 11510 | 11233 | 11527 | 11540 | 11596 |
| Positive control = carbonyl cyanide 3-chlorophenylhydrazone (CCCP), Negative control = No efflux pump inhibitor (NO EPI), an experiment is performed in duplicate (*n*=2) | | | | | | | | | | | | |

| Supp. Table 9: Effect of different concentrations of isoorientin on the accumulation of ethidium bromide in *S. aureus* | | | | | | | | | | | | |
| --- | --- | --- | --- | --- | --- | --- | --- | --- | --- | --- | --- | --- |
| Time  hh:mm:ss | 125 µg/mL | | 62.5 µg/mL | | 31.25 µg/mL | | 15.26 µg/mL | | NO EPI | | CCCP | |
| 0:00:00 | 9566 | 9591 | 9401 | 9423 | 9834 | 9875 | 9801 | 9867 | 9455 | 9461 | 9766 | 9756 |
| 0:05:00 | 9893 | 9882 | 9699 | 9657 | 9998 | 10017 | 9933 | 9979 | 9267 | 9229 | 9995 | 9914 |
| 0:10:00 | 9854 | 9855 | 9615 | 9754 | 9991 | 9942 | 10188 | 10154 | 9264 | 9158 | 10166 | 10119 |
| 0:15:00 | 9919 | 9905 | 9583 | 9418 | 10101 | 10129 | 10043 | 10084 | 9388 | 9384 | 10250 | 10203 |
| 0:20:00 | 10110 | 10187 | 9444 | 9654 | 10214 | 10449 | 10197 | 10122 | 9300 | 9518 | 10168 | 10193 |
| 0:25:00 | 10203 | 10213 | 9761 | 9423 | 10123 | 10171 | 10188 | 10177 | 9268 | 9207 | 10138 | 10152 |
| 0:30:00 | 10119 | 10100 | 9894 | 9807 | 10069 | 10190 | 10136 | 10149 | 9130 | 9303 | 10129 | 10154 |
| 0:35:00 | 10291 | 10242 | 9477 | 9918 | 10049 | 9970 | 10276 | 10229 | 9349 | 9330 | 10118 | 10125 |
| 0:40:00 | 10267 | 10247 | 9702 | 9767 | 10345 | 10320 | 10368 | 10351 | 9395 | 9313 | 10194 | 10120 |
| 0:45:00 | 10211 | 10261 | 9710 | 9512 | 10369 | 10651 | 10487 | 10448 | 9399 | 9263 | 10329 | 10312 |
| Positive control = carbonyl cyanide 3-chlorophenylhydrazone (CCCP), Negative control = No efflux pump inhibitor (NO EPI), an experiment is performed in duplicate (*n*=2) | | | | | | | | | | | | |

| Supp. Table 10: Effect of different concentrations of isoorientin on the accumulation of ethidium bromide in MRSA | | | | | | | | | | | | |
| --- | --- | --- | --- | --- | --- | --- | --- | --- | --- | --- | --- | --- |
| Time  hh:mm:ss | 125 µg/mL | | 62.5 µg/mL | | 31.25 µg/mL | | 15.26 µg/mL | | NO EPI | | CCCP | |
| 0:00:00 | 12477 | 12469 | 12819 | 12807 | 13456 | 13430 | 12889 | 12800 | 12188 | 12178 | 13158 | 13189 |
| 0:05:00 | 13501 | 13868 | 12678 | 12682 | 14199 | 14132 | 13659 | 13552 | 12608 | 12646 | 14479 | 14492 |
| 0:10:00 | 13758 | 13718 | 12998 | 13000 | 14267 | 14231 | 13705 | 13753 | 12758 | 12761 | 14170 | 14123 |
| 0:15:00 | 13883 | 13872 | 13109 | 13113 | 13947 | 13924 | 13901 | 13847 | 12515 | 12638 | 14659 | 14693 |
| 0:20:00 | 14043 | 14096 | 13045 | 13068 | 14187 | 14129 | 14265 | 14341 | 12365 | 12695 | 14750 | 14795 |
| 0:25:00 | 14075 | 14033 | 12899 | 12800 | 14160 | 14109 | 14349 | 14301 | 12892 | 12821 | 14587 | 14533 |
| 0:30:00 | 14137 | 14172 | 12911 | 12965 | 14638 | 14620 | 14185 | 14143 | 12970 | 12964 | 14660 | 14653 |
| 0:35:00 | 13513 | 13539 | 13087 | 13077 | 14109 | 14031 | 14495 | 14459 | 13089 | 13005 | 14538 | 14583 |
| 0:40:00 | 14078 | 14060 | 14056 | 14062 | 14669 | 14603 | 14239 | 14237 | 13165 | 13131 | 13420 | 13459 |
| 0:45:00 | 13819 | 13860 | 13598 | 13574 | 14497 | 14469 | 14250 | 14271 | 13049 | 13039 | 14007 | 14044 |
| Positive control = carbonyl cyanide 3-chlorophenylhydrazone (CCCP), Negative control = No efflux pump inhibitor (NO EPI), an experiment is performed in duplicate (*n*=2) | | | | | | | | | | | | |

| Supp. Table 11: Effect of different concentrations of isoorientin on the accumulation of ethidium bromide in *K. pneumoniae* | | | | | | | | | | | | |
| --- | --- | --- | --- | --- | --- | --- | --- | --- | --- | --- | --- | --- |
| Time  hh:mm:ss | 125 µg/mL | | 62.5 µg/mL | | 31.25 µg/mL | | 15.26 µg/mL | | NO EPI | | CCCP | |
| 0:00:00 | 11540 | 11190 | 11284 | 11352 | 11430 | 11018 | 11110 | 11497 | 11178 | 11276 | 11193 | 11189 |
| 0:05:00 | 11465 | 11616 | 11573 | 11567 | 11640 | 11495 | 11683 | 11623 | 11572 | 11885 | 12093 | 12067 |
| 0:10:00 | 11380 | 11683 | 11902 | 11908 | 11755 | 11715 | 11502 | 11587 | 11707 | 11882 | 12508 | 12214 |
| 0:15:00 | 11790 | 11793 | 12390 | 12321 | 12242 | 12033 | 11510 | 11913 | 11656 | 11849 | 12602 | 12603 |
| 0:20:00 | 11986 | 11876 | 12134 | 12432 | 12193 | 12133 | 11873 | 11837 | 12093 | 12000 | 126302 | 12307 |
| 0:25:00 | 12074 | 12118 | 12773 | 12715 | 12080 | 12415 | 12171 | 12341 | 11998 | 12084 | 12683 | 12673 |
| 0:30:00 | 12019 | 12008 | 12420 | 12465 | 12489 | 12550 | 12335 | 12222 | 11966 | 12133 | 12848 | 12800 |
| 0:35:00 | 12303 | 12340 | 12583 | 12572 | 12483 | 12506 | 12002 | 12406 | 12102 | 12100 | 13094 | 13000 |
| 0:40:00 | 12462 | 12163 | 12774 | 12982 | 12842 | 12522 | 12073 | 12514 | 12281 | 12122 | 13094 | 13001 |
| 0:45:00 | 12537 | 12367 | 12772 | 12799 | 12773 | 12623 | 12576 | 12873 | 12336 | 12178 | 13319 | 13341 |
| Positive control = carbonyl cyanide 3-chlorophenylhydrazone (CCCP), Negative control = No efflux pump inhibitor (NO EPI), an experiment is performed in duplicate (*n*=2) | | | | | | | | | | | | |

| Supp. Table 12: Effect of different concentrations of isoorientin on the accumulation of ethidium bromide in ESBL *K. pneumoniae* | | | | | | | | | | | | |
| --- | --- | --- | --- | --- | --- | --- | --- | --- | --- | --- | --- | --- |
| Time  hh:mm:ss | 125 µg/mL | | 62.5 µg/mL | | 31.25 µg/mL | | 15.26 µg/mL | | NO EPI | | CCCP | |
| 0:00:00 | 39493 | 39409 | 41954 | 41963 | 39886 | 39862 | 39751 | 39744 | 39212 | 39877 | 39654 | 39638 |
| 0:05:00 | 40639 | 40648 | 42634 | 42664 | 41745 | 41765 | 40620 | 40991 | 38677 | 38917 | 40845 | 40867 |
| 0:10:00 | 42293 | 42706 | 43854 | 43830 | 42386 | 42395 | 42191 | 42009 | 38965 | 38901 | 41997 | 41949 |
| 0:15:00 | 42793 | 42789 | 45423 | 45452 | 43945 | 43982 | 43809 | 43824 | 40445 | 40481 | 42976 | 42956 |
| 0:20:00 | 44673 | 44698 | 46633 | 46148 | 45364 | 45382 | 43953 | 43946 | 41499 | 41945 | 44245 | 44240 |
| 0:25:00 | 45593 | 45586 | 47616 | 47403 | 47575 | 47559 | 44934 | 44991 | 43265 | 43279 | 44964 | 44999 |
| 0:30:00 | 46473 | 46477 | 48145 | 48113 | 48145 | 48155 | 46764 | 46792 | 44459 | 44305 | 45756 | 45706 |
| 0:35:00 | 46529 | 46937 | 50087 | 50044 | 48764 | 48750 | 46887 | 46889 | 44954 | 44986 | 47054 | 47085 |
| 0:40:00 | 46843 | 46828 | 49735 | 49738 | 49387 | 49352 | 48689 | 48829 | 45971 | 45817 | 47454 | 47436 |
| 0:45:00 | 47164 | 47196 | 49994 | 49901 | 51323 | 51382 | 50146 | 50170 | 46635 | 46668 | 48334 | 48307 |
| Positive control = carbonyl cyanide 3-chlorophenylhydrazone (CCCP), Negative control = No efflux pump inhibitor (NO EPI), an experiment is performed in duplicate (*n*=2) | | | | | | | | | | | | |

| Supp. Table 13: Effect of different concentrations of orientin on the efflux of ethidium bromide in *E. coli* | | | | | | | | | | | | |
| --- | --- | --- | --- | --- | --- | --- | --- | --- | --- | --- | --- | --- |
| Time  hh:mm:ss | 125 µg/mL | | 62.5 µg/mL | | 31.25 µg/mL | | 15.26 µg/mL | | NO EPI | | CCCP | |
| 0:00:00 | 165233 | 165390 | 163839 | 163673 | 162748 | 162489 | 164749 | 164492 | 163572 | 163492 | 165524 | 165472 |
| 0:05:00 | 162932 | 162301 | 159289 | 159489 | 160834 | 160929 | 159849 | 159821 | 158682 | 158183 | 164582 | 164739 |
| 0:10:00 | 162710 | 162593 | 159289 | 159347 | 158749 | 158940 | 158744 | 158153 | 157621 | 157033 | 164391 | 164738 |
| 0:15:00 | 161912 | 161833 | 158389 | 158949 | 158683 | 158378 | 158849 | 158546 | 157193 | 157031 | 164839 | 164739 |
| 0:20:00 | 161130 | 161290 | 157283 | 157489 | 158949 | 158493 | 158493 | 158849 | 156422 | 156134 | 164329 | 164739 |
| 0:25:00 | 161840 | 161930 | 157830 | 157749 | 158748 | 158774 | 157949 | 157743 | 156923 | 156242 | 162739 | 162724 |
| 0:30:00 | 158930 | 158130 | 157389 | 157749 | 157839 | 157749 | 157649 | 157492 | 156483 | 156481 | 159730 | 159391 |
| 0:35:00 | 159730 | 159830 | 157280 | 157949 | 157802 | 157729 | 156840 | 156749 | 156482 | 156249 | 158842 | 158034 |
| 0:40:00 | 158573 | 158390 | 156383 | 156930 | 156940 | 156782 | 156749 | 156921 | 155722 | 155284 | 157830 | 157648 |
| 0:45:00 | 158930 | 158830 | 157304 | 157849 | 155749 | 155546 | 155748 | 155489 | 154938 | 154163 | 157657 | 157749 |
| Positive control = carbonyl cyanide 3-chlorophenylhydrazone (CCCP), Negative control = No efflux pump inhibitor (NO EPI), an experiment is performed in duplicate (*n*=2) | | | | | | | | | | | | |

| Supp. Table 14: Effect of different concentrations of orientin on the efflux of ethidium bromide in ESBL *E. coli* | | | | | | | | | | | | |
| --- | --- | --- | --- | --- | --- | --- | --- | --- | --- | --- | --- | --- |
| Time  hh:mm:ss | 125 µg/mL | | 62.5 µg/mL | | 31.25 µg/mL | | 15.26 µg/mL | | NO EPI | | CCCP | |
| 0:00:00 | 40344 | 40392 | 37647 | 37234 | 38494 | 38759 | 37749 | 37849 | 35034 | 35940 | 37739 | 37749 |
| 0:05:00 | 38792 | 38648 | 36924 | 36524 | 37249 | 37859 | 34849 | 34578 | 30948 | 30553 | 36567 | 36648 |
| 0:10:00 | 38567 | 38589 | 36749 | 36824 | 35749 | 35053 | 33638 | 33938 | 30124 | 30045 | 36824 | 36849 |
| 0:15:00 | 37840 | 37489 | 36284 | 36284 | 35284 | 35194 | 33493 | 33173 | 30850 | 30749 | 35041 | 35794 |
| 0:20:00 | 37420 | 37490 | 35024 | 35489 | 34749 | 34759 | 33384 | 33738 | 29044 | 29035 | 35958 | 35749 |
| 0:25:00 | 37830 | 37489 | 35840 | 35849 | 34274 | 34950 | 33748 | 33567 | 29552 | 29683 | 34658 | 34739 |
| 0:30:00 | 37749 | 37424 | 35249 | 35378 | 35493 | 35585 | 32274 | 32475 | 29593 | 29733 | 34638 | 34834 |
| 0:35:00 | 36492 | 36480 | 35749 | 35478 | 34384 | 34749 | 32454 | 32824 | 29193 | 29945 | 34042 | 34839 |
| 0:40:00 | 36389 | 36489 | 35924 | 35468 | 34759 | 34658 | 31749 | 31174 | 28920 | 28932 | 34749 | 34739 |
| 0:45:00 | 36480 | 36467 | 34934 | 34470 | 34840 | 34747 | 31394 | 31730 | 28233 | 28830 | 33350 | 33547 |
| Positive control = carbonyl cyanide 3-chlorophenylhydrazone (CCCP), Negative control = No efflux pump inhibitor (NO EPI), an experiment is performed in duplicate (*n*=2) | | | | | | | | | | | | |

| Supp. Table 15: Effect of different concentrations of orientin on the efflux of ethidium bromide in *S. aureus* | | | | | | | | | | | | |
| --- | --- | --- | --- | --- | --- | --- | --- | --- | --- | --- | --- | --- |
| Time  hh:mm:ss | 125 µg/mL | | 62.5 µg/mL | | 31.25 µg/mL | | 15.26 µg/mL | | NO EPI | | CCCP | |
| 0:00:00 | 11209 | 11478 | 11839 | 11904 | 10638 | 10579 | 10113 | 10618 | 9019 | 9618 | 16933 | 16384 |
| 0:05:00 | 9376 | 9384 | 10638 | 10226 | 9734 | 9839 | 9249 | 9761 | 8183 | 8162 | 14739 | 14021 |
| 0:10:00 | 10190 | 10286 | 10930 | 10561 | 9648 | 9386 | 9748 | 9655 | 7628 | 7538 | 14033 | 14572 |
| 0:15:00 | 10369 | 10572 | 10230 | 10131 | 9749 | 9281 | 9542 | 9616 | 6749 | 6102 | 15839 | 15653 |
| 0:20:00 | 9501 | 9529 | 10938 | 10258 | 8830 | 8687 | 10683 | 10160 | 6294 | 6535 | 14633 | 14581 |
| 0:25:00 | 9178 | 9246 | 10472 | 10403 | 8242 | 8515 | 10972 | 10209 | 6739 | 6895 | 15839 | 15153 |
| 0:30:00 | 9793 | 9099 | 9893 | 9294 | 8637 | 8199 | 9743 | 9716 | 7274 | 7101 | 15893 | 15111 |
| 0:35:00 | 8920 | 8502 | 8920 | 8423 | 8930 | 8518 | 8684 | 8823 | 7739 | 7598 | 15733 | 15693 |
| 0:40:00 | 8598 | 8965 | 10781 | 10247 | 7793 | 7645 | 8927 | 8337 | 6830 | 6970 | 14839 | 14140 |
| 0:45:00 | 8091 | 8478 | 9938 | 9474 | 7738 | 7641 | 7534 | 7670 | 6928 | 6595 | 139387 | 13789 |
| Positive control = carbonyl cyanide 3-chlorophenylhydrazone (CCCP), Negative control = No efflux pump inhibitor (NO EPI), an experiment is performed in duplicate (*n*=2) | | | | | | | | | | | | |

| Supp. Table 16: Effect of different concentrations of orientin on the efflux of ethidium bromide in MRSA | | | | | | | | | | | | |
| --- | --- | --- | --- | --- | --- | --- | --- | --- | --- | --- | --- | --- |
| Time  hh:mm:ss | 125 µg/mL | | 62.5 µg/mL | | 31.25 µg/mL | | 15.26 µg/mL | | NO EPI | | CCCP | |
| 0:00:00 | 25746 | 25200 | 24032 | 24988 | 26803 | 26739 | 25840 | 25431 | 24749 | 24965 | 27942 | 27790 |
| 0:05:00 | 24934 | 24345 | 22840 | 22010 | 24440 | 24748 | 24024 | 24532 | 22749 | 22291 | 25934 | 25987 |
| 0:10:00 | 23892 | 23436 | 22830 | 22105 | 24078 | 24848 | 24894 | 24157 | 21840 | 21481 | 26294 | 26453 |
| 0:15:00 | 22940 | 22954 | 21840 | 21770 | 23972 | 23048 | 23935 | 23106 | 20830 | 20398 | 25045 | 25381 |
| 0:20:00 | 21389 | 21496 | 20933 | 20011 | 22871 | 22749 | 23284 | 23148 | 20749 | 20975 | 25242 | 25394 |
| 0:25:00 | 20738 | 20240 | 19749 | 19965 | 21355 | 21553 | 22638 | 22279 | 19824 | 19874 | 24924 | 24791 |
| 0:30:00 | 20749 | 20060 | 19938 | 19456 | 21795 | 21648 | 23749 | 23187 | 18940 | 18907 | 24383 | 24891 |
| 0:35:00 | 19992 | 19671 | 18638 | 18826 | 20146 | 20138 | 22928 | 22680 | 18840 | 18501 | 23193 | 23999 |
| 0:40:00 | 19749 | 19816 | 18733 | 18259 | 19647 | 19359 | 21734 | 21864 | 17835 | 17350 | 22940 | 22425 |
| 0:45:00 | 18193 | 18460 | 17648 | 17784 | 19267 | 19274 | 20739 | 20349 | 17924 | 17515 | 21924 | 21850 |
| Positive control = carbonyl cyanide 3-chlorophenylhydrazone (CCCP), Negative control = No efflux pump inhibitor (NO EPI), an experiment is performed in duplicate (*n*=2) | | | | | | | | | | | | |

| Supp. Table 17: Effect of different concentrations of orientin on the efflux of ethidium bromide in *K. pneumoniae* | | | | | | | | | | | | |
| --- | --- | --- | --- | --- | --- | --- | --- | --- | --- | --- | --- | --- |
| Time  hh:mm:ss | 125 µg/mL | | 62.5 µg/mL | | 31.25 µg/mL | | 15.26 µg/mL | | NO EPI | | CCCP | |
| 0:00:00 | 38749 | 38079 | 34940 | 34430 | 34183 | 34008 | 34729 | 30738 | 34194 | 34522 | 45382 | 45614 |
| 0:05:00 | 36103 | 36928 | 33183 | 33840 | 31739 | 31829 | 34739 | 34739 | 30930 | 30132 | 39839 | 39270 |
| 0:10:00 | 37302 | 37559 | 37092 | 37450 | 37183 | 37362 | 37183 | 37738 | 31829 | 31946 | 40739 | 40595 |
| 0:15:00 | 36930 | 36600 | 35139 | 35054 | 33530 | 33737 | 31739 | 31173 | 30024 | 30400 | 36284 | 36178 |
| 0:20:00 | 33930 | 33223 | 31739 | 31976 | 30813 | 30066 | 31839 | 31499 | 26832 | 26439 | 39247 | 39761 |
| 0:25:00 | 34940 | 34666 | 29299 | 29248 | 33082 | 33185 | 33638 | 33505 | 24849 | 24708 | 34038 | 34708 |
| 0:30:00 | 32930 | 32914 | 30839 | 30278 | 31739 | 31240 | 33904 | 33898 | 27294 | 27003 | 31294 | 31957 |
| 0:35:00 | 37840 | 37105 | 33839 | 33464 | 32104 | 32241 | 36739 | 36389 | 27094 | 27157 | 34839 | 34600 |
| 0:40:00 | 33103 | 33513 | 35739 | 35017 | 35683 | 35936 | 34019 | 34481 | 27385 | 27551 | 33482 | 33755 |
| 0:45:00 | 27893 | 27151 | 31133 | 31251 | 340294 | 34367 | 32893 | 32816 | 27749 | 27708 | 33837 | 33991 |
| Positive control = carbonyl cyanide 3-chlorophenylhydrazone (CCCP), Negative control = No efflux pump inhibitor (NO EPI), an experiment is performed in duplicate (*n*=2) | | | | | | | | | | | | |

| Supp. Table 18: Effect of different concentrations of orientin on the efflux of ethidium bromide in ESBL *K. pneumoniae* | | | | | | | | | | | | |
| --- | --- | --- | --- | --- | --- | --- | --- | --- | --- | --- | --- | --- |
| Time  hh:mm:ss | 125 µg/mL | | 62.5 µg/mL | | 31.25 µg/mL | | 15.26 µg/mL | | NO EPI | | CCCP | |
| 0:00:00 | 81830 | 85637 | 78024 | 78405 | 78124 | 78512 | 71043 | 79031 | 79274 | 72547 | 92802 | 92320 |
| 0:05:00 | 89312 | 82240 | 75391 | 75269 | 76093 | 76617 | 78244 | 77104 | 76394 | 70643 | 89694 | 89990 |
| 0:10:00 | 88303 | 82584 | 75740 | 75846 | 77593 | 77896 | 78324 | 77729 | 68324 | 68255 | 86042 | 88571 |
| 0:15:00 | 87390 | 80500 | 78201 | 76346 | 76740 | 76718 | 76824 | 75372 | 76943 | 71531 | 86004 | 88601 |
| 0:20:00 | 78393 | 78648 | 78301 | 76321 | 72042 | 75532 | 72894 | 76678 | 78024 | 71817 | 86354 | 87280 |
| 0:25:00 | 77933 | 76536 | 77931 | 76828 | 75830 | 75330 | 76824 | 75049 | 78305 | 70105 | 80924 | 88435 |
| 0:30:00 | 76839 | 76141 | 78302 | 74096 | 71038 | 74216 | 76923 | 72827 | 60285 | 69846 | 86835 | 87042 |
| 0:35:00 | 74673 | 74429 | 79313 | 75109 | 79844 | 72786 | 71894 | 71298 | 62057 | 68621 | 86942 | 86521 |
| 0:40:00 | 76829 | 76094 | 77391 | 73374 | 70824 | 70916 | 68492 | 69087 | 60285 | 67317 | 88402 | 86100 |
| 0:45:00 | 79283 | 77967 | 77938 | 72378 | 72940 | 72689 | 68204 | 69046 | 62042 | 66237 | 81904 | 85058 |
| Positive control = carbonyl cyanide 3-chlorophenylhydrazone (CCCP), Negative control = No efflux pump inhibitor (NO EPI), an experiment is performed in duplicate (*n*=2) | | | | | | | | | | | | |

| Supp. Table 19: Effect of different concentrations of isoorientin on the efflux of ethidium bromide in *E. coli* | | | | | | | | | | | | |
| --- | --- | --- | --- | --- | --- | --- | --- | --- | --- | --- | --- | --- |
| Time  hh:mm:ss | 125 µg/mL | | 62.5 µg/mL | | 31.25 µg/mL | | 15.26 µg/mL | | NO EPI | | CCCP | |
| 0:00:00 | 159374 | 155028 | 148688 | 141042 | 157024 | 150932 | 148792 | 148744 | 146844 | 143824 | 155833 | 151712 |
| 0:05:00 | 147024 | 148528 | 143636 | 143636 | 149724 | 148284 | 146243 | 146776 | 146924 | 141604 | 145824 | 148103 |
| 0:10:00 | 142842 | 148537 | 142094 | 147024 | 142804 | 147941 | 149824 | 146365 | 140913 | 141374 | 144924 | 148024 |
| 0:15:00 | 140823 | 146457 | 142724 | 147024 | 141931 | 146839 | 147244 | 145703 | 141702 | 141369 | 147283 | 147704 |
| 0:20:00 | 147302 | 145373 | 141356 | 147924 | 144802 | 145340 | 140244 | 145748 | 141093 | 141373 | 149722 | 147549 |
| 0:25:00 | 141802 | 143478 | 141970 | 145223 | 142924 | 145247 | 141832 | 144772 | 139703 | 139954 | 146944 | 146673 |
| 0:30:00 | 148023 | 142373 | 140041 | 146924 | 147924 | 144533 | 140913 | 143029 | 139635 | 139736 | 140941 | 145390 |
| 0:35:00 | 141949 | 142961 | 140107 | 147024 | 142454 | 144362 | 142047 | 142136 | 140142 | 140474 | 147084 | 146961 |
| 0:40:00 | 148134 | 142028 | 140084 | 148394 | 144024 | 143974 | 146184 | 141955 | 140692 | 140216 | 143583 | 145148 |
| 0:45:00 | 149204 | 141457 | 139317 | 137024 | 148424 | 143428 | 146294 | 140433 | 139274 | 139027 | 142694 | 144457 |
| Positive control = carbonyl cyanide 3-chlorophenylhydrazone (CCCP), Negative control = No efflux pump inhibitor (NO EPI), an experiment is performed in duplicate (*n*=2) | | | | | | | | | | | | |

| Supp. Table 20: Effect of different concentrations of isoorientin on the efflux of ethidium bromide in ESBL *E. coli* | | | | | | | | | | | | |
| --- | --- | --- | --- | --- | --- | --- | --- | --- | --- | --- | --- | --- |
| Time  hh:mm:ss | 125 µg/mL | | 62.5 µg/mL | | 31.25 µg/mL | | 15.26 µg/mL | | NO EPI | | CCCP | |
| 0:00:00 | 12592 | 12754 | 13592 | 13817 | 15692 | 15402 | 15801 | 15494 | 12923 | 12046 | 17972 | 17705 |
| 0:05:00 | 10692 | 10854 | 11592 | 11627 | 12988 | 13218 | 12702 | 12904 | 9132 | 9866 | 16703 | 16192 |
| 0:10:00 | 11083 | 11654 | 11702 | 11587 | 11900 | 12724 | 12575 | 12738 | 9702 | 9625 | 15722 | 15538 |
| 0:15:00 | 10193 | 10444 | 9982 | 10290 | 12081 | 11966 | 11846 | 11418 | 8793 | 8644 | 14822 | 14710 |
| 0:20:00 | 10638 | 10869 | 9539 | 9862 | 11901 | 11438 | 11932 | 11050 | 8632 | 8576 | 14802 | 14303 |
| 0:25:00 | 10173 | 10469 | 9683 | 9814 | 10911 | 10229 | 10101 | 10792 | 8682 | 8419 | 14702 | 14329 |
| 0:30:00 | 9702 | 9263 | 9539 | 9190 | 10702 | 10174 | 9973 | 10140 | 8721 | 8015 | 14802 | 14216 |
| 0:35:00 | 9692 | 9006 | 8572 | 8812 | 9702 | 9892 | 9715 | 9482 | 7702 | 7316 | 13992 | 13955 |
| 0:40:00 | 8692 | 8347 | 9830 | 9958 | 9792 | 9101 | 8613 | 8854 | 6803 | 6606 | 13023 | 13274 |
| 0:45:00 | 7973 | 7815 | 8972 | 8645 | 8893 | 8400 | 8083 | 8886 | 6702 | 6538 | 12902 | 13312 |
| Positive control = carbonyl cyanide 3-chlorophenylhydrazone (CCCP), Negative control = No efflux pump inhibitor (NO EPI), an experiment is performed in duplicate (*n*=2) | | | | | | | | | | | | |

| Supp. Table 21: Effect of different concentrations of isoorientin on the efflux of ethidium bromide in *S. aureus* | | | | | | | | | | | | |
| --- | --- | --- | --- | --- | --- | --- | --- | --- | --- | --- | --- | --- |
| Time  hh:mm:ss | 125 µg/mL | | 62.5 µg/mL | | 31.25 µg/mL | | 15.26 µg/mL | | NO EPI | | CCCP | |
| 0:00:00 | 16755 | 17259 | 13091 | 13441 | 21681 | 21510 | 22791 | 23632 | 13893 | 14580 | 23910 | 23329 |
| 0:05:00 | 17293 | 17010 | 12901 | 12601 | 18091 | 18218 | 19832 | 20706 | 10381 | 11166 | 18782 | 18109 |
| 0:10:00 | 16092 | 16844 | 12901 | 12441 | 18913 | 18100 | 17372 | 17751 | 10893 | 10622 | 16892 | 16691 |
| 0:15:00 | 12343 | 12755 | 11913 | 11214 | 17901 | 17869 | 15091 | 15465 | 9802 | 9152 | 17901 | 17892 |
| 0:20:00 | 11903 | 11310 | 11092 | 11472 | 16809 | 17090 | 12801 | 12318 | 9802 | 9173 | 15489 | 16541 |
| 0:25:00 | 9702 | 9478 | 11802 | 11142 | 16012 | 16783 | 13892 | 13370 | 8902 | 8375 | 15891 | 16984 |
| 0:30:00 | 9093 | 9844 | 11902 | 11043 | 16901 | 16100 | 14183 | 14874 | 7091 | 7159 | 16671 | 17658 |
| 0:35:00 | 9092 | 9755 | 10802 | 10587 | 15912 | 15700 | 13892 | 13167 | 6981 | 6807 | 16801 | 17210 |
| 0:40:00 | 8913 | 8310 | 9802 | 9500 | 14821 | 14752 | 11801 | 11949 | 5190 | 5790 | 15910 | 15613 |
| 0:45:00 | 8093 | 8478 | 98024 | 9000 | 13792 | 13869 | 11267 | 11905 | 5981 | 5134 | 15091 | 15262 |
| Positive control = carbonyl cyanide 3-chlorophenylhydrazone (CCCP), Negative control = No efflux pump inhibitor (NO EPI), an experiment is performed in duplicate (*n*=2) | | | | | | | | | | | | |

| Supp. Table 22: Effect of different concentrations of isoorientin on the efflux of ethidium bromide in MRSA | | | | | | | | | | | | |
| --- | --- | --- | --- | --- | --- | --- | --- | --- | --- | --- | --- | --- |
| Time  hh:mm:ss | 125 µg/mL | | 62.5 µg/mL | | 31.25 µg/mL | | 15.26 µg/mL | | NO EPI | | CCCP | |
| 0:00:00 | 38920 | 39305 | 34893 | 35886 | 35823 | 36873 | 26167 | 26429 | 30872 | 30992 | 27165 | 28198 |
| 0:05:00 | 32289 | 33267 | 30124 | 30921 | 26892 | 27959 | 24981 | 24043 | 23092 | 23494 | 24792 | 25992 |
| 0:10:00 | 31209 | 32632 | 28782 | 28864 | 23792 | 24275 | 24981 | 24566 | 21672 | 21953 | 24980 | 25001 |
| 0:15:00 | 31892 | 31669 | 26712 | 27419 | 24895 | 25297 | 23093 | 23972 | 21082 | 21973 | 24891 | 24395 |
| 0:20:00 | 28781 | 28669 | 24781 | 25941 | 22091 | 22201 | 22123 | 22174 | 19845 | 20145 | 24981 | 24931 |
| 0:25:00 | 27543 | 27646 | 23891 | 23733 | 23389 | 23024 | 22982 | 22687 | 19910 | 19790 | 24793 | 24439 |
| 0:30:00 | 24782 | 25478 | 22129 | 22374 | 21092 | 21338 | 21783 | 21642 | 18457 | 18658 | 22550 | 23950 |
| 0:35:00 | 22389 | 23321 | 21091 | 21532 | 20124 | 20114 | 20835 | 20923 | 17182 | 17837 | 22463 | 23443 |
| 0:40:00 | 23656 | 23478 | 20781 | 20733 | 20673 | 20733 | 20674 | 20517 | 17283 | 17060 | 20942 | 21992 |
| 0:45:00 | 21782 | 22321 | 20981 | 20532 | 20783 | 20482 | 19872 | 19271 | 16382 | 16714 | 22382 | 22534 |
| Positive control = carbonyl cyanide 3-chlorophenylhydrazone (CCCP), Negative control = No efflux pump inhibitor (NO EPI), an experiment is performed in duplicate (*n*=2) | | | | | | | | | | | | |

| Supp. Table 23: Effect of different concentrations of isoorientin on the efflux of ethidium bromide in *K. pneumoniae* | | | | | | | | | | | | |
| --- | --- | --- | --- | --- | --- | --- | --- | --- | --- | --- | --- | --- |
| Time  hh:mm:ss | 125 µg/mL | | 62.5 µg/mL | | 31.25 µg/mL | | 15.26 µg/mL | | NO EPI | | CCCP | |
| 0:00:00 | 33453 | 34754 | 33102 | 33597 | 40982 | 39671 | 29103 | 29515 | 33980 | 33761 | 29345 | 29658 |
| 0:05:00 | 30989 | 30631 | 29891 | 30395 | 34891 | 34245 | 28720 | 28932 | 26952 | 27953 | 28912 | 28615 |
| 0:10:00 | 27980 | 28812 | 29081 | 29644 | 34091 | 34187 | 27820 | 28613 | 27091 | 27353 | 28901 | 28100 |
| 0:15:00 | 28682 | 28450 | 27980 | 28950 | 30172 | 30937 | 26091 | 26399 | 27172 | 27604 | 27763 | 27282 |
| 0:20:00 | 28091 | 28300 | 28091 | 28702 | 29891 | 30738 | 25782 | 26747 | 27823 | 28854 | 27615 | 27153 |
| 0:25:00 | 27120 | 27754 | 26893 | 27055 | 30901 | 30152 | 26890 | 27459 | 26489 | 27439 | 27395 | 27839 |
| 0:30:00 | 26091 | 26046 | 26920 | 26644 | 30102 | 30878 | 26563 | 26399 | 26783 | 26371 | 25980 | 26212 |
| 0:35:00 | 26722 | 26107 | 24093 | 25950 | 30091 | 30878 | 25378 | 25613 | 25123 | 25252 | 26873 | 26658 |
| 0:40:00 | 25801 | 25686 | 24890 | 25055 | 29901 | 29274 | 24302 | 24932 | 24834 | 24439 | 26763 | 26041 |
| 0:45:00 | 25368 | 25865 | 24682 | 24702 | 28891 | 28187 | 24873 | 24399 | 23912 | 23737 | 25234 | 25631 |
| Positive control = carbonyl cyanide 3-chlorophenylhydrazone (CCCP), Negative control = No efflux pump inhibitor (NO EPI), an experiment is performed in duplicate (*n*=2) | | | | | | | | | | | | |

| Supp. Table 24: Effect of different concentrations of isoorientin on the efflux of ethidium bromide in ESBL *K. pneumoniae* | | | | | | | | | | | | |
| --- | --- | --- | --- | --- | --- | --- | --- | --- | --- | --- | --- | --- |
| Time  hh:mm:ss | 125 µg/mL | | 62.5 µg/mL | | 31.25 µg/mL | | 15.26 µg/mL | | NO EPI | | CCCP | |
| 0:00:00 | 19803 | 20282 | 19210 | 20512 | 20012 | 20946 | 21674 | 21195 | 19878 | 20326 | 25883 | 25811 |
| 0:05:00 | 18901 | 18105 | 19584 | 19305 | 20123 | 19989 | 21123 | 21402 | 18987 | 18319 | 24654 | 24996 |
| 0:10:00 | 18345 | 18628 | 20091 | 20217 | 21920 | 21268 | 21870 | 21111 | 17980 | 17865 | 24784 | 24440 |
| 0:15:00 | 18980 | 18278 | 18892 | 18666 | 20102 | 20303 | 21901 | 21144 | 17231 | 17855 | 23124 | 23763 |
| 0:20:00 | 17324 | 17400 | 18982 | 18647 | 19802 | 20031 | 20981 | 20521 | 16468 | 16246 | 22946 | 22527 |
| 0:25:00 | 16287 | 16416 | 18796 | 18230 | 18234 | 18865 | 18923 | 19463 | 15091 | 15812 | 21980 | 22141 |
| 0:30:00 | 16123 | 16544 | 16902 | 16605 | 20192 | 19403 | 18012 | 18723 | 15980 | 15218 | 23980 | 23400 |
| 0:35:00 | 16542 | 16686 | 18012 | 18641 | 19324 | 18529 | 18902 | 18078 | 15870 | 15261 | 23232 | 23120 |
| 0:40:00 | 15980 | 15387 | 17860 | 18877 | 19921 | 19150 | 17611 | 17701 | 14123 | 14521 | 22987 | 22679 |
| 0:45:00 | 15109 | 15354 | 18163 | 18842 | 20506 | 20852 | 17901 | 17546 | 14674 | 14583 | 23980 | 23030 |
| Positive control = carbonyl cyanide 3-chlorophenylhydrazone (CCCP), Negative control = No efflux pump inhibitor (NO EPI), an experiment is performed in duplicate (*n*=2) | | | | | | | | | | | | |


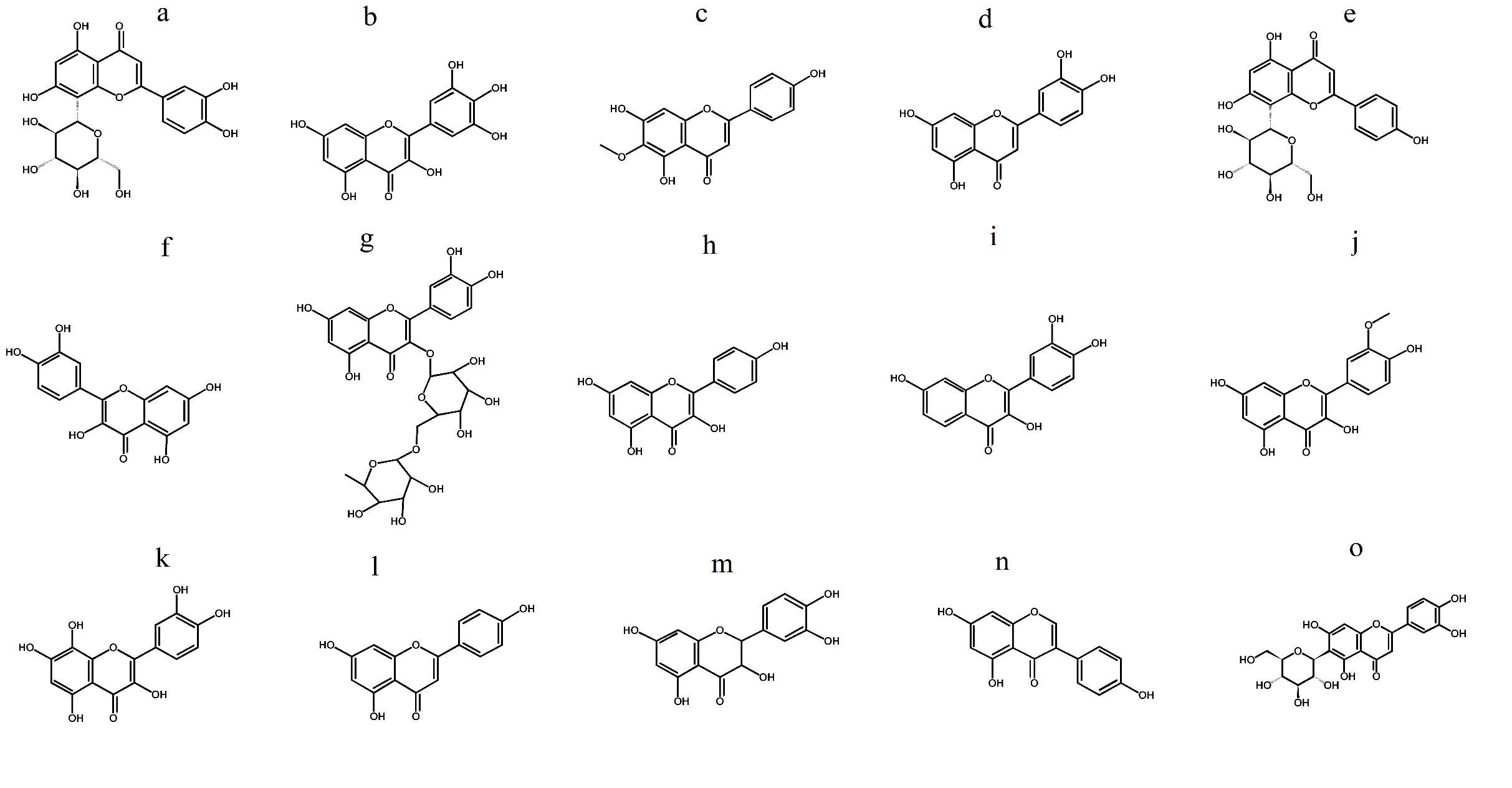
Supp. Figure 1: Chemical structure of orientin (6a), myricetin (6b), hispidulin (6c), luteolin (6d), vitexin (6e), quercetin (6f), rutin (6g), kaempferol (6h), fisetin (6i), isorhamnetin (6j), gossypetin (6k), apigenin (6l), taxifolin (6m), genistein (6n), and isoorientin (6o)
